# Dynamics of Intact MexAB-OprM Efflux Pump: Focusing on the MexA-OprM Interface

**DOI:** 10.1038/s41598-017-16497-w

**Published:** 2017-11-28

**Authors:** Cesar A. López, Timothy Travers, Klaas M. Pos, Helen I. Zgurskaya, S. Gnanakaran

**Affiliations:** 1 Theoretical Biology and Biophysics Group, Los Alamos National Laboratory, Los Alamos, New Mexico 87545 United States; 2 Center for Nonlinear Sciences, Los Alamos National Laboratory, Los Alamos, New Mexico 87545 United States; 3 Institute of Biochemistry, Goethe University, Frankfurt am Main, Germany; 4 Cluster of Excellence Frankfurt, Goethe University, Frankfurt am Main, Germany; 5 Department of Chemistry and Biochemistry, University of Oklahoma, 101 Stephenson Parkway, Norman, Oklahoma 73019 United States

## Abstract

Antibiotic efflux is one of the most critical mechanisms leading to bacterial multidrug resistance. Antibiotics are effluxed out of the bacterial cell by a tripartite efflux pump, a complex machinery comprised of outer membrane, periplasmic adaptor, and inner membrane protein components. Understanding the mechanism of efflux pump assembly and its dynamics could facilitate discovery of novel approaches to counteract antibiotic resistance in bacteria. We built here an intact atomistic model of the *Pseudomonas aeruginosa* MexAB-OprM pump in a Gram-negative membrane model that contained both inner and outer membranes separated by a periplasmic space. All-atom molecular dynamics (MD) simulations confirm that the fully assembled pump is stable in the microsecond timescale. Using a combination of all-atom and coarse-grained MD simulations and sequence covariation analysis, we characterized the interface between MexA and OprM in the context of the entire efflux pump. These analyses suggest a plausible mechanism by which OprM is activated via opening of its periplasmic aperture through a concerted interaction with MexA.

## Introduction

Successful antibiotic therapies have been severely compromised by the spread of antibiotic resistance in bacterial pathogens^1–4^. Multidrug efflux transporters—an innate and powerful resistance mechanism—are capable of extruding a number of chemically unrelated antimicrobials from a bacterial cell and enable the survival of bacteria in noxious environments^5–14^. *Pseudomonas aeruginosa* is an opportunistic pathogen that causes severe infections in humans. Its inherent resistance to diverse clinically important antibiotics and its ability to acquire high-level resistance makes infections caused by this bacterium particularly difficult to treat^15–19^. As with other Gram-negative bacteria, the multidrug resistance (MDR) of *P*. *aeruginosa* mainly results from the synergism between the low-permeability barrier of the outer membrane and the expression of drug efflux pumps^20–29^.

**Figure 1:**
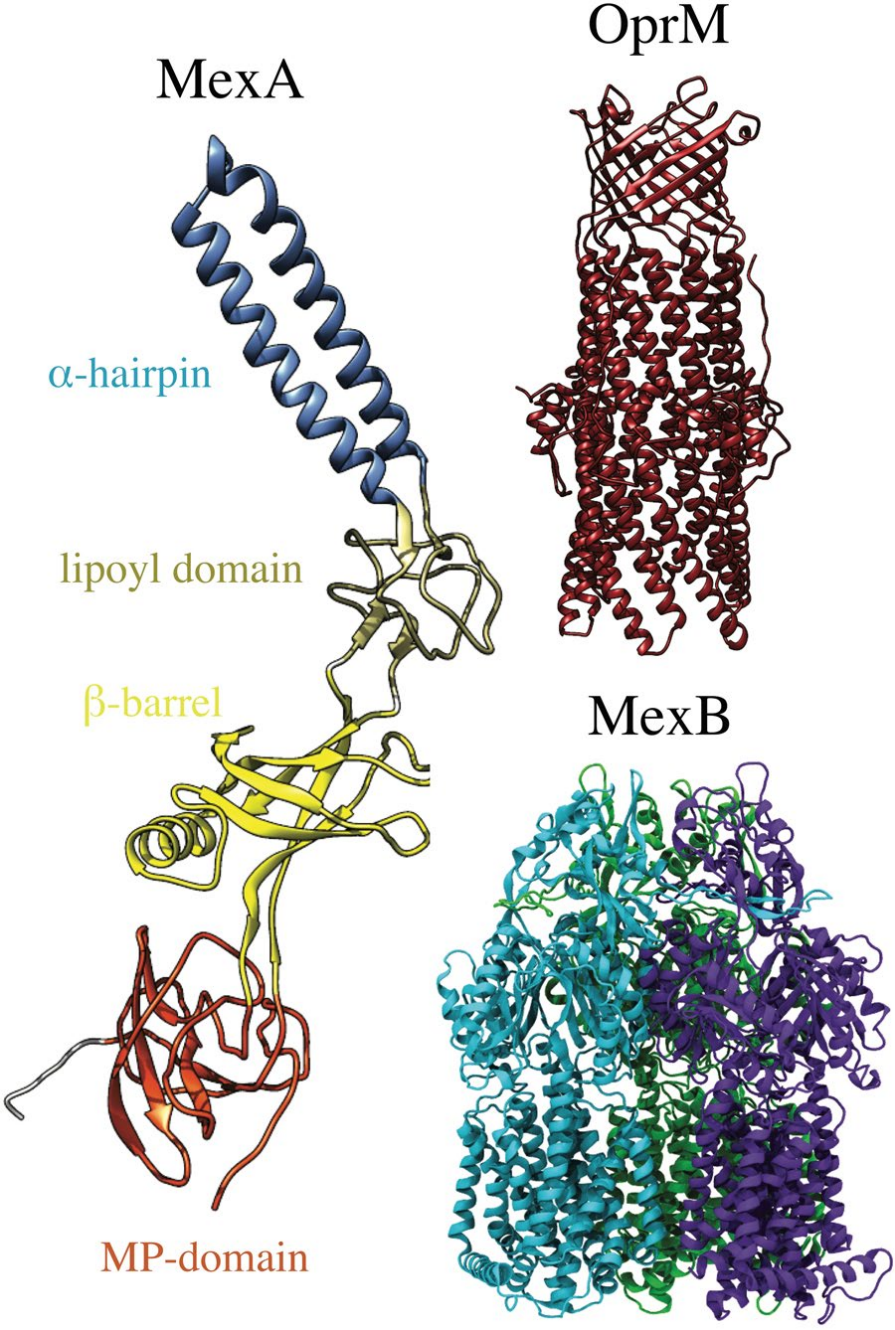
Protein components of *P*. *aeruginosa* MexAB-OprM efflux pump. The overall topology of each pump component is shown in terms of their individual domains. MexA (left) is the periplasmic adaptor in this pump, and its domains are highlighted here using different colors for the α-hairpin (light blue), lipoyl (tan), β-barrel (yellow) and membrane-proximal or MP (orange) domains. For clarity, only a single protomer of the MexA hexamer is shown. The OprM trimer (top-right) is the outer membrane channel in this pump, and is shown with all three protomers in red. The MexB trimer (bottom-right) is the inner membrane transporter in this pump, and individual protomers are colored differently here (cyan, purple, and green).

Previous structural and functional analyses showed that the clinically important efflux pumps of *P*. *aeruginosa* and other Gram-negative bacteria consist of an inner membrane protein component belonging to the Resistance Nodulation cell Division (RND) superfamily of secondary transporters, a channel-forming outer membrane factor (OMF), and a periplasmic membrane fusion protein (MFP)^30–34^. These three components are believed to form a protein complex that extends across both the inner and the outer membranes, allowing the extrusion of drugs from the periplasm directly into the extracellular milieu^34–37^. MexAB-OprM is the major efflux pump of *P*. *aeruginosa* that contributes to clinical antibiotic resistance^17,19–21,30^. In this pump, the proteins MexA, MexB, and OprM act as the MFP, RND transporter, and OMF, respectively (Fig. 1).

MexA and MexB are thought to form a stable complex at the inner membrane, which is recruited by OprM localized in the outer membrane. In addition to MexAB, OprM can functionally interact with different RND transporter/MFP complexes from *P*. *aeruginosa*. This promiscuity of OprM is similar to that of the outer membrane factor TolC from *Escherichia coli*, which in addition to recruiting AcrAB (a close homolog of MexAB) interacts with other various exporters (e.g., AcrAD and AcrEF (RND), EmrAB (Major Facilitator Superfamily or MFS), MacAB (ATP-Binding Cassette Superfamily or ABC)) involved in antibiotic efflux and protein secretion. In contrast, other homologs of OprM such as *P*. *aeruginosa* OprN or OpmH are more discriminative and interact with specific complexes MexEF and TriABC, respectively, but not with MexAB^38–40^.

The extensively studied *E*. *coli* AcrAB-TolC system serves as a structural template for studies of RND-type efflux pumps from Gram-negative bacteria^41–43^. In addition, high-resolution crystal structures of tripartite assembly and efflux pump components from different bacteria facilitate functional and mechanistic analyses of these complexes^32,35,44–49^. Despite low sequence conservation, in general, between corresponding components of efflux pumps from different bacterial species, the major structural features are highly conserved. MexA is a typical MFP with an elongated boomerang-like structure (Fig. 1) that extends from the outer leaflet of the inner membrane deep into the periplasm to meet the OMFs^22,50,51^. This rather linear structure comprises four characteristic domains. Starting at the inner membrane, the domains are the membrane proximal (MP), β-barrel, lipoyl, and α-helical hairpin domains. While these domains are arranged in a linear manner, the N-terminal and C-terminal halves of the protein fold in upon each other to complete the structure. The domains are linked by flexible unstructured regions which give the overall protein its conformational flexibility and allow for a high degree of movement believed to be important for functional interactions with transporters and OMFs. The functional unit of MFPs is a hexamer that assemble as dimers, in which each MFP protomer forms a distinct interaction interface with an RND and an OMF subunit. Trimerization of the MexA dimers results in an inverted funnel-like structure.

As a typical representative of RND transporters, MexB is embedded into the inner membrane by a bundle of twelve transmembrane α-helices (transmembrane segments or TMS), comprised by 2 × 5 TMS parallel repeats (and two coupling helices, TMS2 and TMS8) of which the pseudosymmetric TMS4 and TMS10 form a titratable side chain network at the core of the two bundles. The large periplasmic loops between TMS1 and TMS2 and between TMS7 and TMS8 form a periplasmic domain, the site of substrate recognition (Fig. 1).

Extending from the extracellular space well into the periplasm, OprM is a ~140 Å long, cannon-shaped protein made up of a 12-stranded β-barrel, a hollow cylindrical domain consisting of 12 α-helices, and a mixed α/β equatorial domain (Fig. 1). The β-barrel of trimeric OprM is embedded into the outer membrane, where each protomer contributes only a third of the β-strands. The α-domain is made up of two long helices that span nearly 100 Å into the periplasm, and four shorter helices that stack up to make two long pseudo-continuous helices spanning the length of the entire α-domain as well. The α-helices in combination with the β-barrel domain form a hollow structure, accessible from the extracellular compartment.

The functional unit of RND transporters is a trimer formed through a tight association of the periplasmic part into a two-tier structure of the porter and docking (or funnel) domains. The hexameric funnel of the MFP interacts with both the porter and docking domains of the RND transporters. The large periplasmic α-domain extension of the outer membrane channel is thought to be a docking site for MFPs and transporters. A current model of the assembled pump depicts the MFP as a funnel-like hexameric structure acting as a bridge between the RND transporter and the OMF^52^. In this model, the periplasmic domains of MFP and OMF interact with each other in a tip-to-tip manner and form a continuous open conduit from the OMF-docking subdomain of the transporter and into the external medium. This model is based on recent electron microscopy studies^10,33,35,53,54^. The dynamic assembly of the complex has been studied *in vitro* by surface plasmon resonance and isothermal titration calorimetry techniques, which have allowed for the evaluation of the affinity between components and suggested multi-step sequential models for assembly of the complex^9,55^. Further biochemical studies also suggested that the interactions between pump components are highly dynamic and involve large conformational changes in MFPs^9,10,46^.

In a fully assembled RND pump, the passages and channels are expected to be clear of obstructions to facilitate the transport of drugs^53,54,56^. However, in the available crystal structures the periplasmic entrance of OprM and other OMFs appear to be tightly closed. Moreover, reconstituted TolC has been shown to be impermeable to solutes as small as ions unless a substantial voltage is applied over the membrane^57^. For the tripartite RND-MFP-OMF assembly to be active, the periplasmic gate region is expected to open by association with the inner membrane complex consisting of the RND and MFP components^58–60^. Although the critical assembly step has been successfully reconstituted *in vitro* ^35,37^, the mechanism of OMF recruitment and opening remains unclear. The dense packing of the three curved sets of coiled-coils has been proposed to keep the periplasmic opening of OprM in its “closed” conformation^49^, however a plausible mechanism for transport has been recently proposed based on a cryo-EM structure of the fully assembled pump^56,61^.

In this study, we constructed an atomistic model of MexAB-OprM based on experimental data and probed its dynamics through molecular dynamics (MD) simulations. MD simulations have proven to be an excellent tool to describe the molecular interactions of relevance in biomolecular systems, and to provide insights for processes on the nanosecond to microsecond timescales that are difficult to probe experimentally. Previous MD simulations have been used to analyze the conformational dynamics of individual components of efflux pumps^43,62–68^. These studies provided insights into the conformational flexibility of MFPs, the significant stability of the closed conformation of OMFs, and the transition states of transporters^43,63–66,69^. Here, we provide an atomistic model of the MexAB-OprM pump in a Gram-negative membrane mimic that contains both inner and outer membranes separated by a periplasmic space. MD simulations showed that the fully assembled tripartite pump is stable in the microsecond timescale. Furthermore, we used both all-atom and coarse-grained MD simulations to characterize the interactions of the MexA-OprM interface in the context of the fully assembled pump. Structural rearrangements observed at this interface suggest that the concerted interaction between OprM and MexA initiates the opening of the OprM periplasmic aperture. Sequence covariation analysis between MexA and OprM indicated that the interacting residue pairs identified from our structural models appear to be evolutionary coupled. Finally, a spatial characterization of the entire drug translocation conduit (from the docking domain of the MexB transporter, via the MexA hexameric and OprM α-domain and β-barrel conduit, and to the external medium) is provided along with the thermodynamic profile of drug translocation through this conduit.

## Results

### Construction and dynamics of intact MexAB-OprM pump of *P*. *aeruginosa* in a Gram-negative membrane model

**Figure 2:**
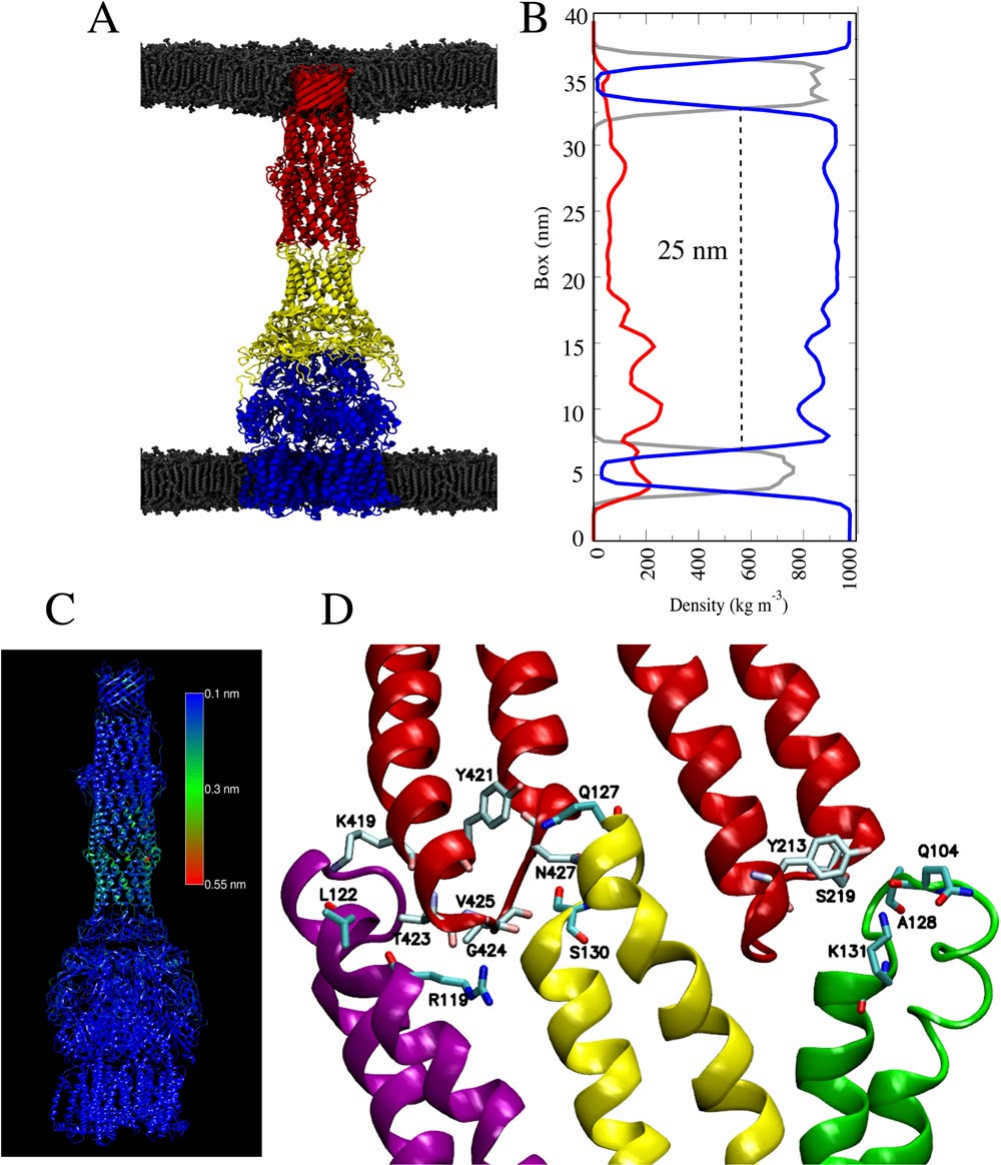
Molecular dynamics simulation of the fully assembled MexAB-OprM efflux pump. (**A**) The tripartite pump (red: OprM, yellow: MexA, blue: MexB) was embedded in two POPC bilayers (gray lipids) to mimic the double membrane bilayers of Gram-negative bacteria. For clarity, water molecules are not shown. (**B**) Mass density profile of the simulated system highlighting the peaks corresponding to the protein structure (red), the POPC lipids (gray), and the water molecules (blue). The density highlights the thickness of the periplasmic region as denoted by the distance between the phosphate groups of the corresponding inner leaflets (25 nm). (**C**) Root mean square fluctuations (RMSF) of the backbone atoms of the whole pump, highlighting higher fluctuations in the contact region between the periplasmic and the outer membrane domains. Fluctuations mostly range between 0.2 and 0.4 nm. (**D**) Close-up of the residues interacting between the periplasmic adaptor and outer membrane proteins during the MD simulation. Note that a single OprM monomer (red ribbons) is able to interact with three periplasmic protomers (green, yellow, and purple ribbons).

The building of an initial fully assembled atomistic model of MexAB-OprM of *P*. *aeruginosa* from its individual components was based on a previously published data-driven approach^70^. To avoid steric clashes, this assembled pump model was subsequently energy minimized and fitted to a 16-Å cryo-EM map of the *E*. *coli* AcrAB-TolC pump using the MDFF approach^71–73^, as shown in Fig. 2. This MD simulation-based cryo-EM fitting approach has been previously applied to reconstruct large protein complexes with reasonable accuracy^72–74^. Upon comparison of initial and final (after 10^6^ fitting steps) configurations, we observed two main changes at the end of the cryo-EM fitting. First, the contact regions between OprM and MexA were biased towards an OprM conformation with an open periplasmic aperture. This is shown as the area enclosed within residues Leu429 located in the tips of the periplasmic helices (Figure S1C, bottom insets). The MDFF fitting of the preassembled MexAB-OprM structure led to changes in the α-hairpins of MexA as well as the periplasmic helices of OprM, accounting for the observed density in this region in the experimental cryo-EM map (Figure S1B). Indeed, the fitting placed the interacting MexA-OprM helices in a cogwheel-like structure, properly stabilized by a series of inter-protein hydrogen bonds as well as hydrophobic interactions, as shown in the contact matrices provided in Figure S2. Interestingly, the conformation adopted by the MexA hexamer α-hairpin helices was similar to that observed for the AcrA hexamer in a recent high-resolution cryo-EM structure of drug-bound AcrAB-TolC^56^ (coordinates in PDB 5NG5) (Figure S3), even though there were no bound drugs to MexAB-OprM during the MDFF fitting. The second change was in the hexameric arrangement of MexA lipoyl and β-domains, as observed in the 16-Å cryo-EM map. Indeed, the original arrangement of heptameric protomers with a spiral assembly that was observed in the X-ray structure (PDB 1VF7) was forced to adopt a configuration satisfying a six-fold symmetry, as shown in the cryo-EM density map^54^. The resulting model accounted for ~95% of the density (if the densities accounting for detergent are omitted). Overall, the resulting MexAB-OprM conformation was used as an initial model for further MD simulations in the context of a simulated Gram-negative outer and inner membrane environment.

We carried out a one-microsecond all-atom MD simulation to evaluate the dynamics and stability of the MexAB-OprM model built from the MDFF approach as described above. This all-atom model describes the pump as the inner membrane MexB transporter in assembly with hexameric MexA and the latter connected to the outer membrane channel OprM (Fig. 2). This arrangement suggests that the interaction of OprM with a MexA hexamer adopting a drug-bound-like conformation should activate the extrusion mechanism by specifically opening the periplasmic aperture of OprM. Initially placed in a double bilayer (mimicking the Gram-negative cell envelope arrangement), the pump obtained after the MDFF fitting retains most of its structure throughout the total simulation time. We measured the inner-membrane/outer-membrane distance in order to calculate the periplasmic space distance (Fig. 2B), and the computed distance between the respective headgroups of lipids constituting the leaflets of the outer and inner membrane facing the periplasmic region gives a value of 25 nm. This value is in very good agreement with the experimental distance (23.9 nm) from cryo-EM pictures of *P*. *aeruginosa* PAO1^75^. As shown in Fig. 2C, most of the structural changes observed in the complex during the MD simulation are localized within the contact points between OprM and MexA. Those fluctuations range from 0.1 to 0.5 nm and remained within that range during the entire simulation. Computed interaction energies (Coulomb + van der Waals) among OprM, MexA and MexB components exhibit only mild fluctuations during the entire microsecond timescale (Figure S4). A detailed inspection of this region indicates which residues contribute to the overall stability of the binding interface between OprM and MexA (Fig. 2D).

### Sequence covariation analysis of the MexA-OprM interface

**Figure 3:**
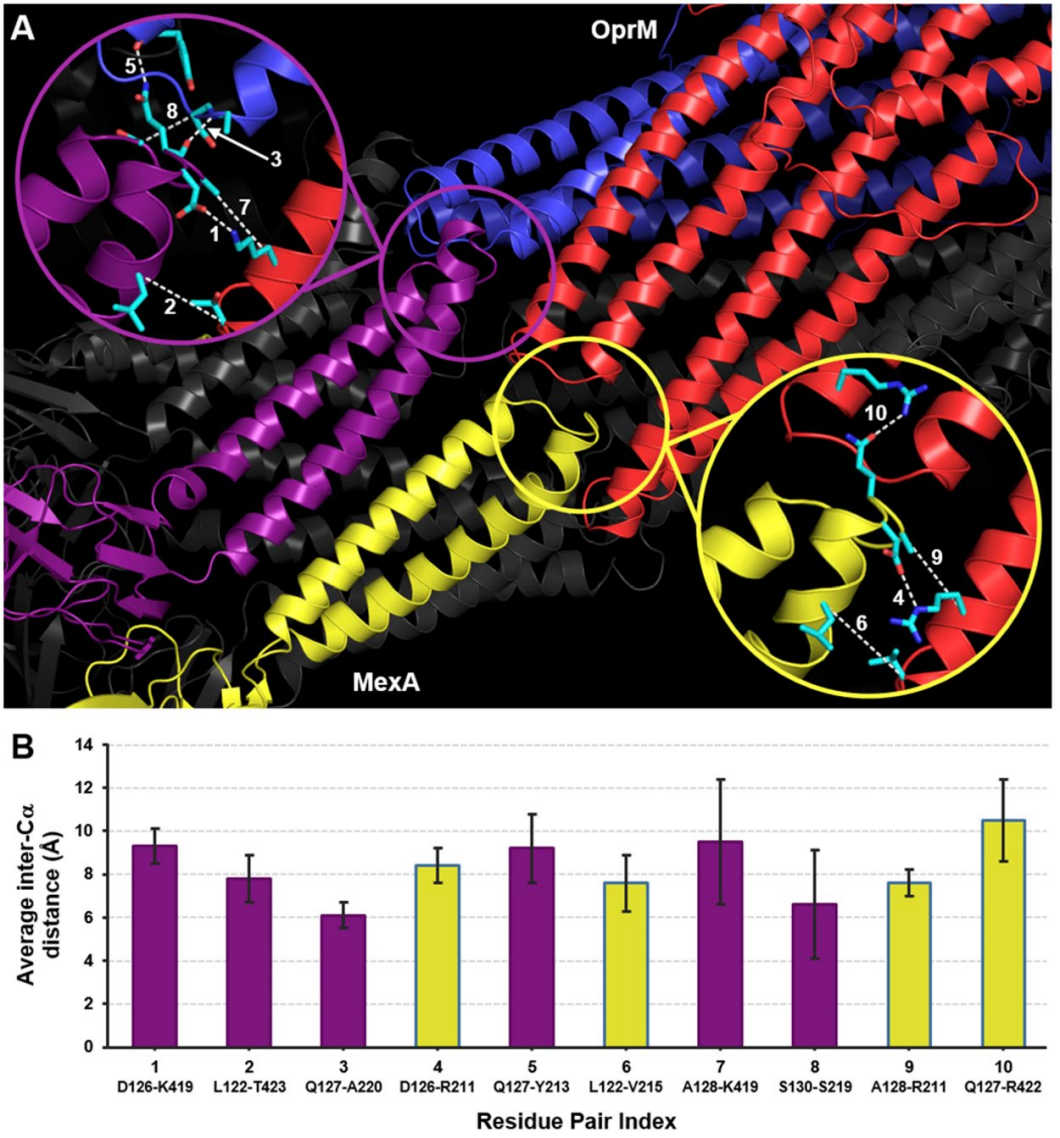
The top ten predicted residues pairs from sequence covariation analysis are found at two adjacent interfaces between MexA and OprM. (**A**) One interface (top-left inset) involves the α-hairpin of a MexA monomer (violet) intercalating between two α-hairpins from separate OprM monomers (blue and red). The other interface (bottom-right inset) involves the α-hairpin of a MexA monomer (yellow) intercalating between two α-hairpins from the same OprM monomer (red). Six of the ten predicted residue pairs occur in the former interface, while the other four are found in the latter interface. Numbers in both insets refer to the GREMLIN ranking of each residue pair (see Table 1). For this snapshot, dashed lines show: (i) SC-SC contacts for pairs 1, 4, and 10; (ii) SC-BB contacts for pairs 3, 5, and 8; and (iii) inter-Cα distances for pairs 2, 6, 7, and 9. (**B**) Average inter-Cα distances for each of the ten residue pairs over all three protomers in the last 500 ns of the 1-μs MD simulation of MexAB-OprM. Residue pairs are colored either violet or yellow depending to which of the two interfaces shown in (**A**) these belong. Error bars give s.d.

The cryo-EM fitting using the MDFF approach showed the interacting hairpin motifs from MexA and OprM forming a cogwheel-like structure, similar to that seen in the adaptor-bridging model for the AcrA-TolC interface^53,56^. To further evaluate the MexA-OprM interface and in particular the interacting residue pairs between them, we performed a sequence covariation analysis for these two proteins. Two online tools for performing this type of analysis, GREMLIN^76^ and EVComplex^77^, were used to identify residue pairs that show covariation with family members of MexA and OprM. Application of both methods to bacterial protein complexes with experimentally-solved structures have shown that both algorithms can reliably identify residue pairs that are near each other at known binding interfaces, indicating that the identified covarying pairs are also predicted to be interacting. For MexA and OprM, both tools predict the same set of ten covarying and interacting residue pairs (Table 1). The residue pairs with ranks 1–4 and 6 are ranked the same by both methods, while the rankings for the other five pairs differ. Interestingly, these ten residue pairs are all found near the cogwheel-like MexA-OprM binding interface for the fitted MexAB-OprM model from earlier (Fig. 3A). As a control, we also performed sequence covariation analysis of MexB and OprM, two components that do not directly interact with each other, using GREMLIN. We found that the identified MexB-OprM residue pairs were all low-confidence predictions, with the top five predictions having GREMLIN scores (interaction probabilities) of 0 (Table S1).

**Table 1:** Top ten predicted covarying residue pairs between MexA and OprM by GREMLIN and EVComplex. Gremlin scores are expressed as probabilities that predicted residue pairs are covarying and interacting, with values ≥ 0.70 giving high-confidence predictions. For EVComplex, the scores are not expressed as probabilities, and values > 0.8 are predicted to be co-varying and interacting. Values in parentheses show the ranking of each residue pair by either method.

MexA residue	OprM Residue	Score (Rank) GREMLIN	Score (Rank) EVComplex
D126	K419	0.99 (1)	3.65 (1)
L122	T423	0.98 (2)	3.08 (2)
Q127	A220	0.95 (3)	2.79 (3)
D126	R211	0.95 (4)	2.63 (4)
Q127	Y213	0.90 (5)	2.16 (7)
L122	V215	0.78 (6)	2.26 (6)
A128	K419	0.76 (7)	2.33 (5)
S130	S219	0.74 (8)	1.46 (9)
A128	R211	0.68 (9)	1.15 (10)
Q127	R422	0.65 (10)	1.66 (8)

Six of the predicted MexA-OprM residue pairs, including the top three predictions, are found at the interface where a MexA hairpin intercalates between two hairpins from adjacent OprM monomers **(**Fig. 3A, top-left inset). The other four residue pairs are found at the interface where a MexA hairpin intercalates between two hairpins from the same OprM monomer (Fig. 3A, bottom-right inset). We next analyzed these ten residue pairs from the last 500 ns of the 1-μs MD simulation of MexAB-OprM, and found that the top nine pairs (based on GREMLIN ranking, used in the rest of the text) have cumulative average inter-Cα distances of less than 10 Å (Fig. 3B). This value is often defined as the cutoff value for initial assessment of potential interactions between two residues^78^.

The tenth-ranked residue pair, MexA Q127 and OprM R422, showed an average inter-Cα distance of 10.2 Å that is just slightly greater than this cutoff. The side chain center of mass (SC-COM) distances were also measured for these residue pairs from the final 500 ns of the 1-μs MD simulation, and the pairs ranked 1–6 and 9 showed average SC-COM distances less than 6.5 Å (Figure S5A,B). This value corresponds to the first peak in the radial distribution function (RDF) between residues in the protein interior^79^, and is often used as the cutoff value for searching potential side chain contacts. Pairs 1 (D126-K419) and 4 (D126-R211) showed the lowest average SC-COM distance values, which is consistent with their formation of salt bridges (see Fig. 3A, top-left inset for pair 1 and bottom-right inset for pair 4). The residue pairs ranked 7 (A128-K419) and 8 (A130-S219) showed cumulative average SC-COM distances slightly greater than the 6.5-Å cutoff. However, breaking the cumulative averages for these two pairs into separate averages for each of the three instances that can occur in the full structure showed that two of these instances had average values below the cutoff, while the third instance had a larger average value that pulled the cumulative average greater than the cutoff. We note that pairs 7 and 8 formed backbone H-bond contacts in the MD simulation (see Fig. 3A, top-left inset), thereby accounting for why both pairs in MexA-OprM satisfied the inter-Cα cutoff but do not consistently satisfy the SC-COM cutoff. The tenth-ranked residue pair showed cumulative and instance-separate SC-COM averages greater than 6.5 Å, indicating that the side chains of MexA Q127 and OprM R422 are not interacting. The bottom-right inset of Fig. 3A shows a frame from the MD simulation where both residues are interacting through an H-bond between their side chains, but this interaction was very short-lived and in fact both side chains were pointing away from one another for essentially most of the trajectory.

### Mechanistic aspects of the role of OprM for functionality of the intact pump

One of the key observations from the MD simulation of the intact pump was that the rearrangement and optimization of the MexA-OprM interface leads to eventual opening of the OprM channel. The MDFF procedure that fitted the all-atom model to the cryo-EM density forced the model towards a conformation where OprM was open. However, the mechanism that leads to opening of the OprM aperture at the molecular scale is not yet well understood. Therefore, we carried out a series of independent simulations on a series of systems from isolated OprM to the OprM-MexA complex in order to elucidate the mechanistic details of OprM aperture opening and the eventual functional activation of the whole pump.

**Figure 4:**
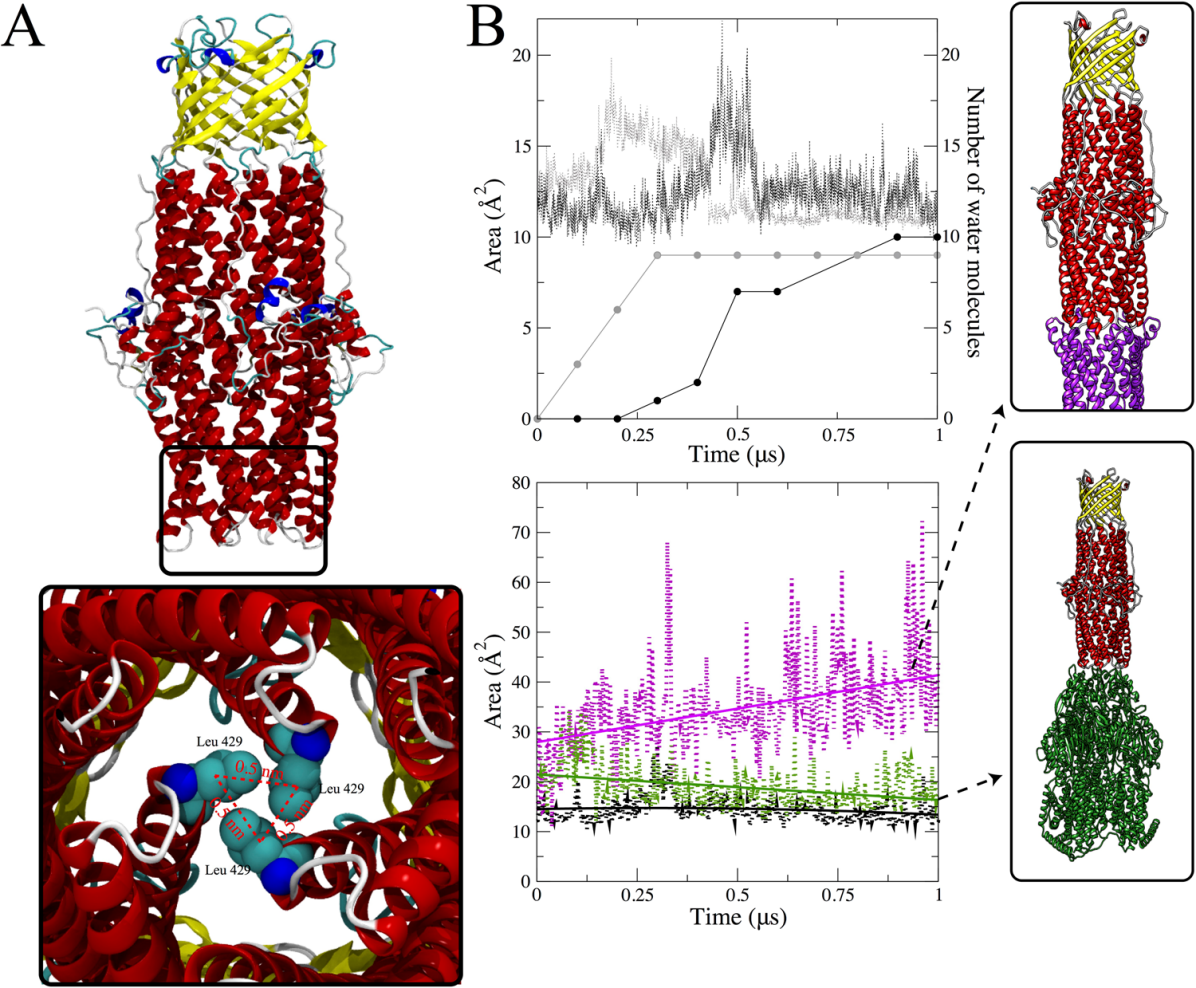
MexA-dependent opening of the OprM periplasmic aperture. (**A**) Structure of OprM highlighting the constraints in the periplasmic tips. Three helices are packed together and enclose three hydrophobic leucine residues (Leu429; one per OprM protomer) with an α-carbon distance value of 0.5 nm between each pair of leucines. These three leucine residues are used as reference points for computing a triangular-shaped area (dotted red lines) for monitoring the aperture area. (**B**) Top panel: Spontaneous partial opening that leads to water translocation through the closed state of OprM. Two independent simulations (black and grey dotted lines) reveal stochastic partial opening with an increase of ~5 Å^2^ that allows the permeation of 10 water molecules. This process occurs within 1 µs of the simulation (black and grey dotted lines). Bottom panel: OprM aperture opening is activated by the close interactions between the outer membrane and periplasmic proteins (purple). The aperture opening was computed as a function of the enclosed triangular shaped area as given in (**A**). As a reference, the average area of closed OprM (black) is also provided. Aperture opening of OprM is not fully triggered by the interaction with the inner component MexB (green) and remains only in a partially opened state.

First, we considered the MD simulations of OprM by itself in a lipid-water system without any other components of the efflux pump. As probed by our simulations, the resting state of an isolated OprM trimer is characterized by virtually no translocation of water molecules through its periplasmic region. A spatially tight clustering of residues at the periplasmic tip of the channel leads to the closed state of the OprM. In fact, a previous report showed that a hydrogen bond network and a cluster of hydrophobic residues take part in this closure^80^. To better probe the conformational aspects of the closed state, we calculated the area enclosed by the α-carbons of the leucine residues located in the sealed end of the channel (Fig. 4A). On average, these residues enclose a triangular ~15 Å^2^ area, which is mostly filled by the leucine residues (shown as spheres in the bottom of Fig. 4A). We found that passage of water molecules through this narrow area exhibits a very low rate (0.01 water molecules per nanosecond). Even in a closed state, OprM can partially open in a stochastic manner to allow the translocation of water molecules (Fig. 4B, top panel). This partial opening is characterized by an increase of the enclosed area by 5 Å^2^. A second simulation using different initial velocities revealed that the partial opening event is stochastic. Nevertheless, the increase in area and the number of water molecules translocated through the duct are in agreement between the two simulations (Fig. 4B, top panel).

Next we considered non-equilibrium MD simulations to explore whether or not an isolated OprM channel would close spontaneously when started from an opened state. The opening of OprM was instituted by imposing a deformation of the periplasmic helices, after which the simulation was started. This simulation shows that the helices are prone to come back together, as indicated by a steady decrease of the aperture area (Figure S6, black line). The full re-establishment of the closed state, however, may occur beyond the microsecond scale and not observed in our simulations. We also probed the relevance of the residues responsible for maintaining the closed state of OprM^49^. Here, we manually incorporated *in silico* mutations at positions Y413F, D433A, and R436G, and simulated from the closed state of OprM for 1 µs. Three main features were found after analyzing the simulation. i) The simulation shows one more opening event, which translates into more waters diffusing per nanosecond (Figure S6, red line). ii) In particular, the aperture area seems to be more expanded (~20 Å^2^ larger) during the opening, which also contributes to more water molecules being translocated at a given time. iii) The mutated OprM is also able to properly seal the terminal tip in the closed state. From our 1-µs simulation, the native OprM remains fully sealed for ~0.8 µs, while the mutated OprM shows a sealed period of ~0.6 µs. Even though this comparison was made with just a single simulation, it may potentially support mutational strategies in the sealing region to modulate the channel opening, either by enhancing the opened or closed states. Combining all these results, we conclude that OprM by itself may not able to remain in an opened state for a prolonged period of time without the assistance of a molecular partner for its activation.

In order to explore the mechanism by which OprM is forced towards the opened state in the intact efflux pump, we docked the closed OprM structure with the equilibrated MexA hexamer structure from the fully assembled pump obtained earlier by MDFF fitting. The details of the system set-up can be found in the Supporting Methods. As soon as this simulation starts, we observed a constant increase of the aperture area, which steadily rises along the microsecond simulation time. As shown in Fig. 4B (bottom panel, purple line), the aperture area reaches ~40 Å^2^, and allows for the passage of small molecules. As a comparison, we also probed whether or not the interactions with the inner membrane MexB is able to trigger the opening of the OprM structure. The evolution of the aperture is given in Fig. 4B (bottom panel, green line). Initiated by a transient perturbation of the periplasmic helices, the area enclosed by the leucine residues steadily drops until reaching values comparable to the partially opened state. The behavior was observed in two independent simulations, thus suggesting that specific molecular interactions with MexA are critical for opening the OprM channel.

**Figure 5:**
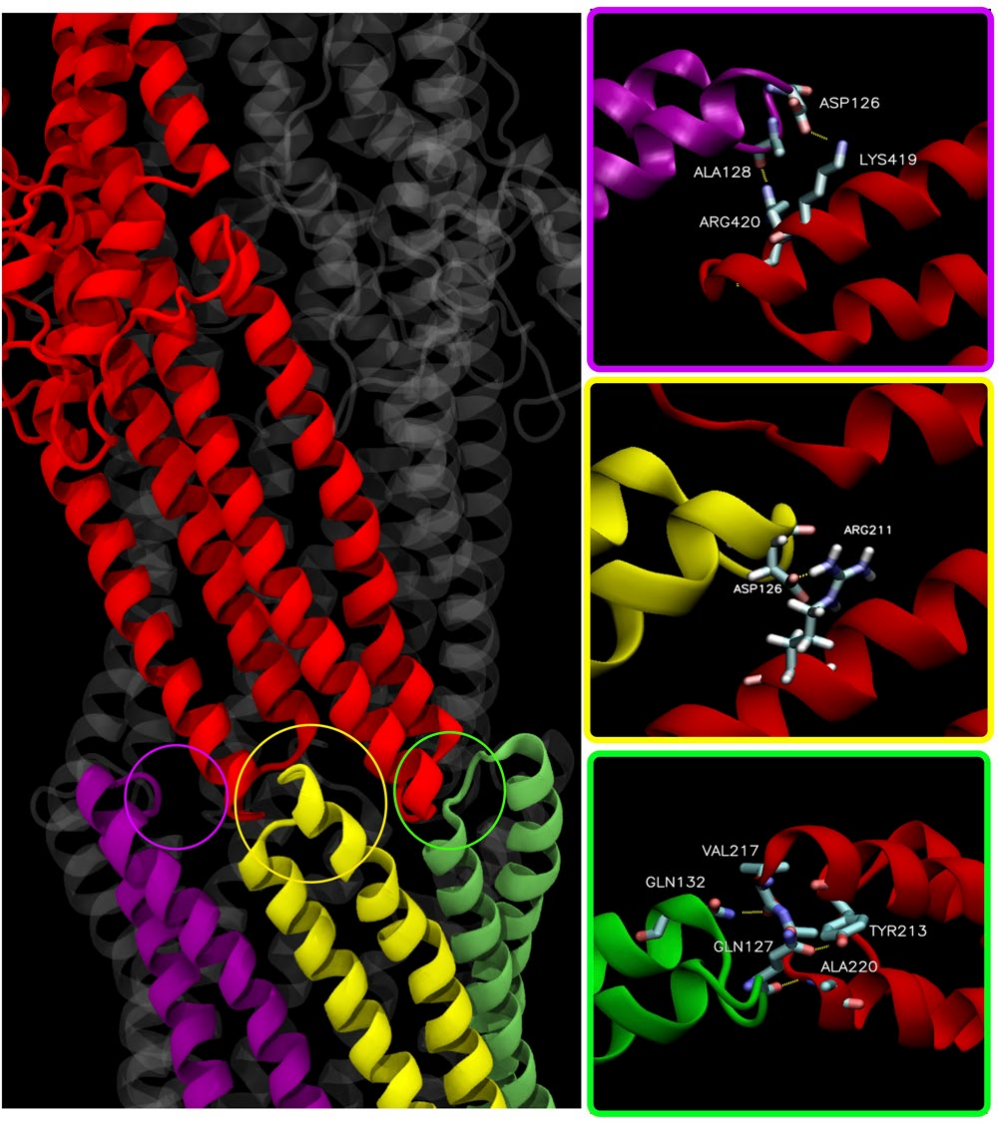
Close-up of the interactions formed during the MexA-dependent opening of the OprM periplasmic aperture. A series of interactions between the α-hairpins of OprM and MexA were formed during the simulation, which are highlighted here for a single OprM protomer (red ribbons). Within these interactions, we present the ones stabilized by specific hydrogen bonds. Thus, two hydrogen bonds are observed with MexA protomer 1 (purple ribbon), one hydrogen bond with MexA protomer 2 (yellow ribbons) and three hydrogen bonds with MexA protomer 3 (green ribbons). Water molecules were removed here for a clearer depiction.

We closely inspected the molecular interactions responsible for the MexA-dependent opening of the OprM periplasmic aperture. Several key interactions are shown in Fig. 5 and independently highlighted for one monomer of the OprM structure (red ribbons). Notably, a single protomer of OprM is able to interact with three monomers of MexA. The same interactions were observed symmetrically distributed for the two other OprM protomers. We counted at least six inter-domain hydrogen bonds formed during the simulation (Fig. 5, insets). Four of these hydrogen bonding pairs (D126-K419, D126-R211, Q127-Y213, and Q127-A220) were captured in the sequence covariation analysis (see Fig. 3). Another pair (A128-R420) was adjacent to one of the predicted covarying pairs (A128-K419). Other interacting residues are reported in the contact maps using a threshold of 0.65 nm (Figure S7 **)**. Interestingly, the contact map obtained from the unbiased simulation resembles very well the contact map computed after the MDFF fitting, supporting the notion that the OprM opening mechanism is mediated by the MexA α-hairpins. The contacts presented in Fig. 5 are in general observed for all three OprM protomers, and show an average SC-COM distance of less than 6.5 Å (Figure S8A,B).

**Figure 6:**
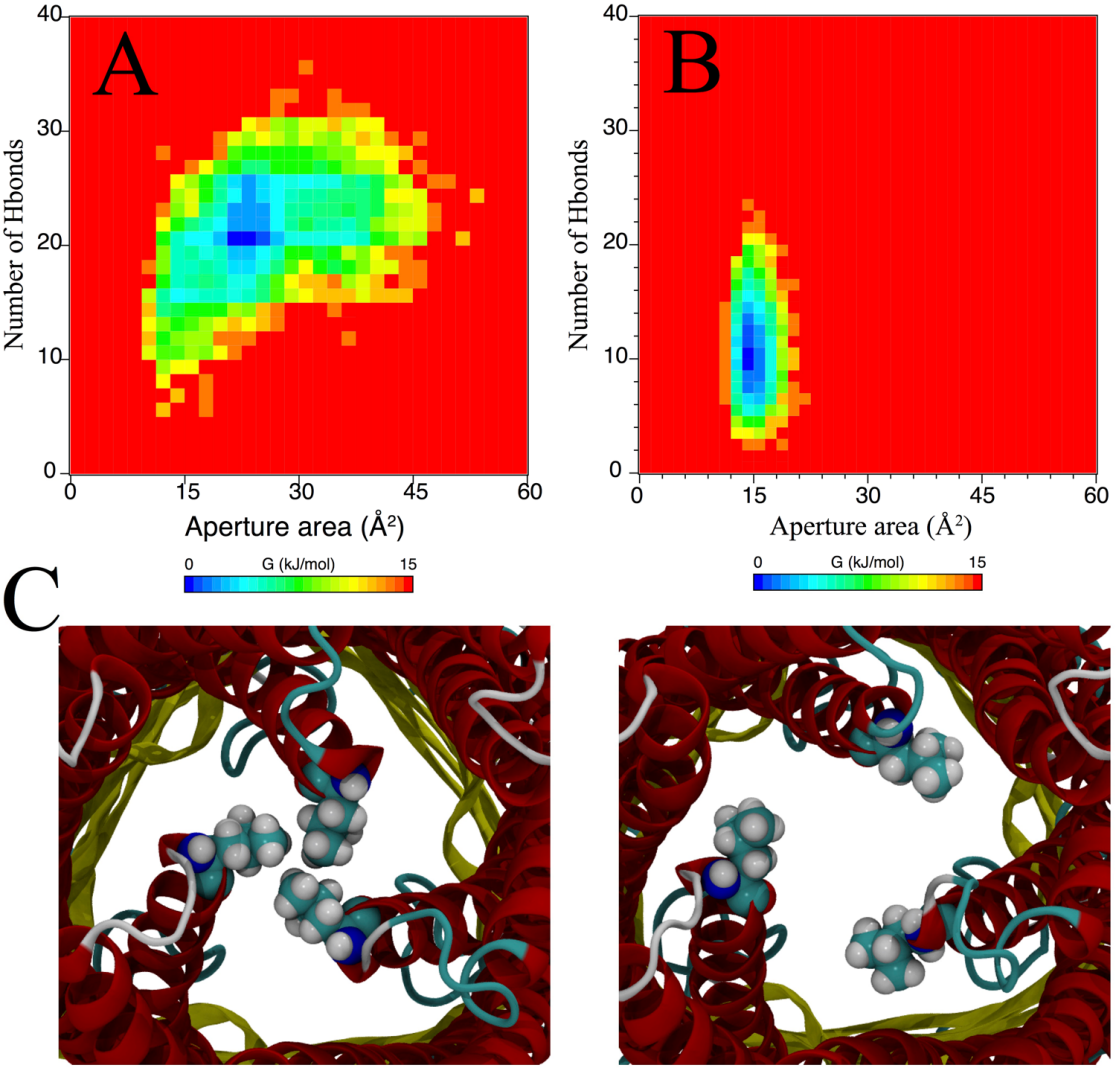
Energy profiles for the opening of the OprM periplasmic aperture. (**A**) Computed energy profile as a function of the number of hydrogen bonds and the aperture area of OprM. This profile featured the aperture opening as triggered by the interaction with MexA. (**B**) Idem as (**A**), however the profile was obtained from the interaction between OprM and the inner component MexB. (**C**) Snapshots featuring the structural mechanism of OprM aperture opening as obtained from (**A**).

At this juncture, we probed whether opening of the OprM aperture could also be facilitated by a direct interaction between MexB and OprM, as proposed by some studies in the *E*. *coli* equivalent AcrB/TolC^8,81^. We compared the dependency of the OprM aperture with respect to the number of hydrogen bonds formed with either MexA or MexB. Figure 6 plots free energy profiles as a function of the aperture area and the number of hydrogen bonds formed with either MexA (Fig. 6A) or MexB (Fig. 6B). The interaction of OprM with MexA covers a wide distribution of aperture areas (ranging from ~15 to ~50 Å^2^). Similarly, there is a progressive increase in the amount of hydrogen bonds formed between both components with increasing aperture area. In contrast, the interaction between OprM and MexB does not show high variations of the channel aperture, and in fact the values are very comparable with OprM in the resting state (i.e., closed state). A very interesting feature, however, suggests that OprM may be able to interact with MexB as shown by the total number of hydrogen bonds coupling both domains. Visualization of the trajectory highlighted in Fig. 6A reveals that the transition between closing and opening of OprM is basically dependent on the swing of helix H8, spanning residues 411–446. Overall, these results suggest that the interaction of OprM with the MexA hexamer adopting a drug-bound-like conformation not only affects the structure of OprM, but also decreases the activation energy required for the opening of the OprM periplasmic aperture.

**Figure 7:**
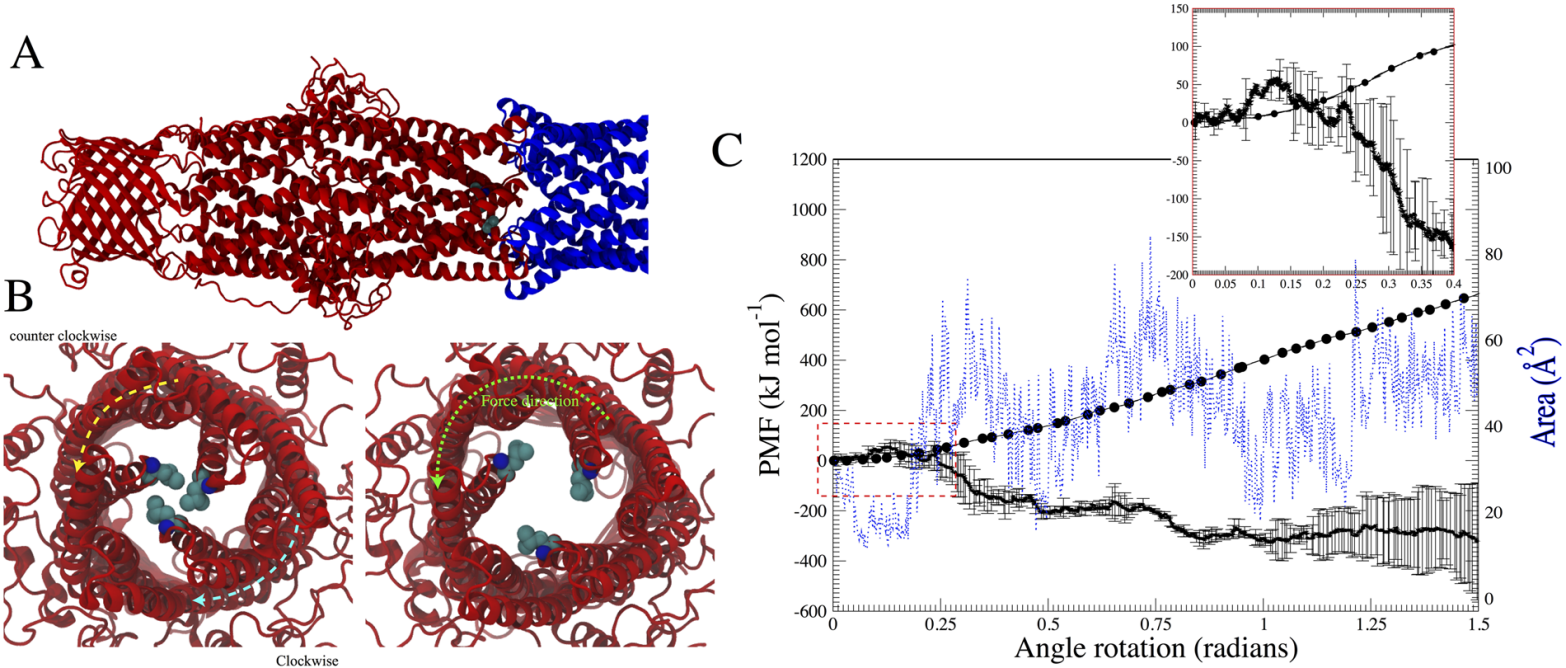
Enforced rotational aperture opening of OprM. (**A**) System set-up highlighting the OprM domain (red ribbons) and the periplasmic protein helices (blue ribbons). The rotational force was applied to the periplasmic protein component. (**B**) Different directions of the force applied in respect to the periplasmic tips in the OprM domain. The counter clockwise rotation is featured by the increase of the area enclosed by the Leu429 (see Results). (**C**) Potential of mean force (PMF) as a function of rotated angle (radians) in the periplasmic domain MexA. Cumulative energy for the counter-clockwise rotation is shown as a black solid line. The small inset highlights the unfavorable process within the first 0.15 radians and is characterized by an increase of ~50 kJ mol^−1^. The rotation becomes favorable after 0.25 radians and is featured by a constant increase of the aperture area (dashed blue line) as shown in the right axis. The clockwise rotation, however, is not favorable as shown by the constant increase in energy with increased rotation angle (black dotted line).

Given that the concerted interaction with MexA is functionally relevant for opening the OprM channel in an intact efflux pump, we evaluated whether our observations are consistent with a different mechanism for the MexA-OprM interface proposed in the literature. Phan *et al*.^80^ suggested an alternative model for the aperture opening of OprM. This mechanism involves an internal rotation of the periplasmic region in an iris-like motion. By using normal mode analysis, they determined that the rotation is coupled with a subtle enlargement of the domain. To analyze the mechanism, we applied an enforced rotation to MexA. Starting from the configuration shown in Fig. 7A, a constant force with a torque of 500 kJ mol^−2^ was applied to the α-hairpin domains of MexA. A snapshot of the trajectory is given in Fig. 7B, for the rotation of ~20 degrees (see Movie S1 that highlights the amino acid rearrangements underlying the iris-like motion). As clearly seen, the Leu429 amino acids of OprM are not fully in contact, thereby resembling a quasi-open state. To more thoroughly describe the opening mechanism through the rotational force, we computed the potential of mean force (PMF) along the rotated angle. Here, we should clarify that the excess of force applied over the system can contribute to an excess of work over the OprM transition. We do not have any proof that our enforced rotational approach is in the regime of an equilibrium process, and so the results provided by the PMF should be treated more as a qualitative outcome.

The initial state (Fig. 7C, small inset and black line) is characterized by a slow increase of the free energy, reaching a barrier of ~50 kJ mol^−1^ in less than 0.15 radians of rotation. However, after this state is reached, the energy continuously drops, suggesting a favorable process. At this point, the first increase of the aperture area is observed (right axis, dashed blue line), jumping from 15 Å^2^ to approximately 50 Å^2^. Keeping the counterclockwise rotation increases the aperture area; however, a close inspection of the simulation clearly shows partial unfolding of the coiled-coil domains of OprM with increased rotation. We also probed the clockwise rotation (Fig. 7C, black circles), and found that the process becomes very high in energy, clearly related to steric clashes occurring from the collisions of the helices containing the leucine residues. Based on these results, we suggest that a slight rotation of OprM may favorably contribute to the opening of the channel.

### Translocation of an antibiotic molecule through the continuous open conduit of OprM in an intact MexAB-OprM pump

**Figure 8:**
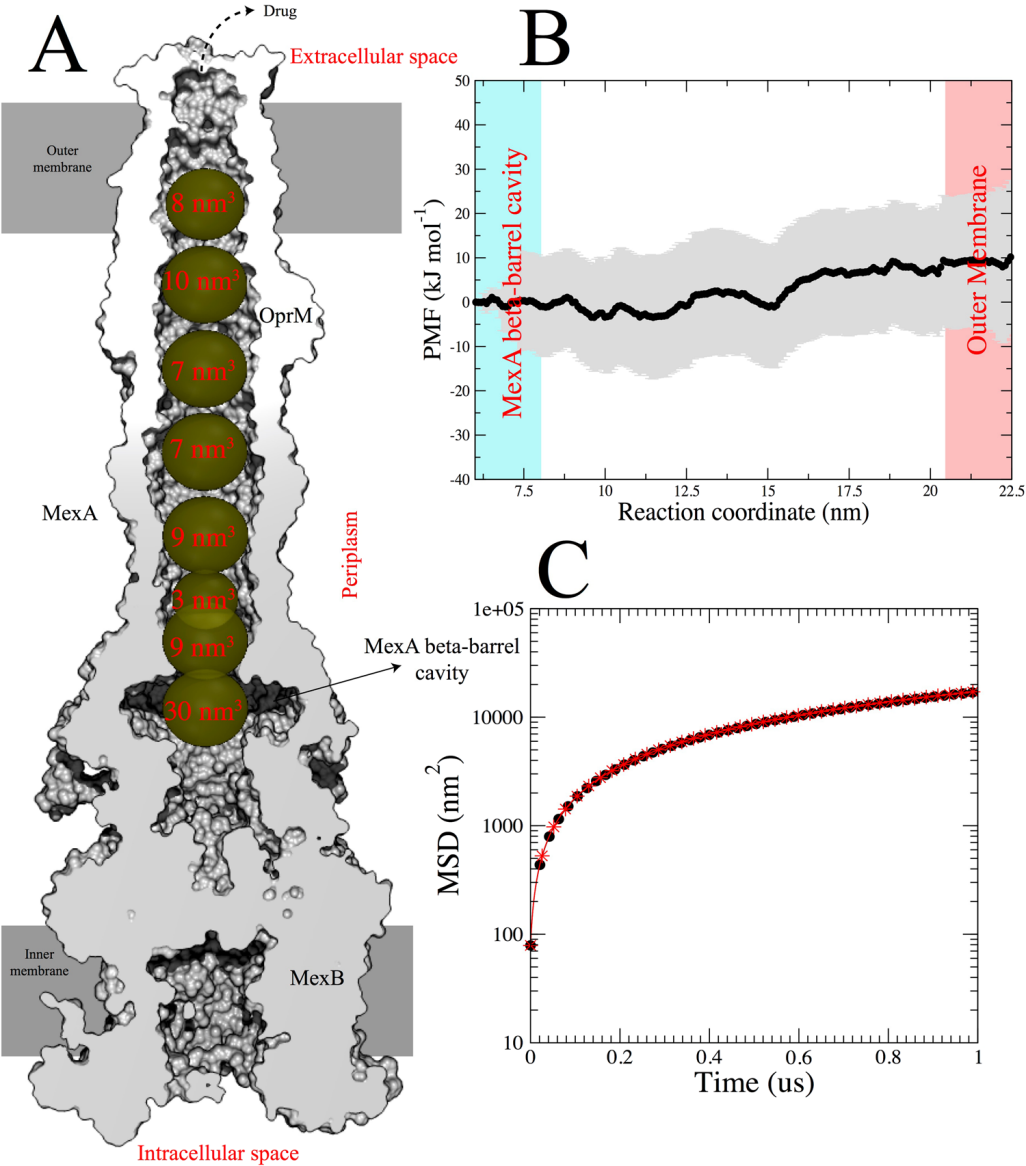
Rifampicin translocation through the conduit of OprM in the MexAB-OprM pump. (**A**) Cross section of the equilibrated fully assembled pump, showing the averaged volume of the conduit along the OprM and MexA complex. (**B**) Potential of mean force for the translocation of rifampicin through the channel formed by the MexA and OprM domains. The total cumulative energy suggests the process is diffusive as values range within thermal fluctuations (~4 kJ mol^−1^). (**C**) Comparison of the mean square displacement (MSD) for the water molecules running inside the OprM-MexA channel (black circles) and water molecules outside the boundaries of the pump (red stars), suggesting that water diffusion inside the pump is not affected.

The evidence presented above suggests that interactions with MexA hexamer in a drug-bound-like conformation can open the OprM channel and allow the passage of an antibiotic through the channel to the external medium. We next probed the nature of the OprM channel to explore how the interior topology of that channel could affect drug translocation. Figure 8A shows the average cross-section of the fully assembled MexAB-OprM pump as obtained during the MD simulation. The bilayers are depicted as gray rectangles. Through this view, it is possible to visualize a conduit that is open to the bacterial cell exterior and traverses from the transmembrane β-barrel of OprM, through the OprM periplasmic domain to the hexameric periplasmic assembly of MexA and continues all the way to the receptor region in MexB (docking domain). The cross-section also reveals that the channel is sealed to leakage along the periplasmic region with no gaps between OprM and MexA. Accordingly, this conduit seems to be the only exit pathway for the efflux of substrates, which will be delivered into this chamber through the successive LTO transitions and peristaltic movements of the inner membrane component^68^.

We tracked the diffusion of the water molecules within the pump channel to probe how well the efflux pump is sealed. The leakage of water at the interfaces was tracked by collectively measuring the diffusion of water molecules already placed inside the funnel region. We found that no molecules traveled outside the boundaries of the channel, as the major component of the diffusion runs on the normal of the channel. This result suggests that molecules traveling inside the channel (e.g. drugs) will only be allowed to travel vertically through OprM and will be spatially restricted by the channel diameter.

Next, we probed the translocation of a single rifampicin molecule through the assembled pump. Although there are currently no experimentally reported permeation rates for this drug in *P*. *aeruginosa* and no specific channels or porins have so far been implicated for its permeation, we chose to study this drug here in order to show that a large drug can freely translocate through the channel of the assembled pump. Given the complexity and enormous number of particles in the system, we carried out coarse-grained MD simulations that can provide relevant dynamical information with reasonable computational effort. The starting configuration places the drug within the central cavity formed by MexA hexamer on the interface with MexB and assumes that no proton motive force is required for the final extrusion. As depicted in Fig. 8B, for full translocation the drug should cover a distance of 15 nm. At first glance, the funnel like structure formed by MexA and OprM does not pose any steric impedance. This is supported by measurements of the inner volume in different regions of the funnel. As shown in Fig. 8A, the entire funnel provides enough space with inner volumes ranging from 3 to 10 nm^3^. The narrowest regions were localized at the hexameric constriction formed by the MexA lipoyl domains and at the beginning of the α-helical hairpins. The MexA β-barrel cavity, on the other hand, shows a volume of 30 nm^3^ and is able to host a maximum of ~40 rifampicin molecules. However, given the size of rifampicin (0.8 nm^3^), only a few of them may be able to pass into the funnel at a given period of time.

The potential of mean force for translocation of a rifampicin drug molecule through the tunnel is presented in Fig. 8B. The total energy buildup corresponds to ~8 kJ mol^−1^ (from the MexA β-barrel cavity to the extracellular compartment), suggesting that this translocation is a spontaneous process. Indeed, visual inspection of the process shows that the drug is able to interact with several residues inside the channel, but these interactions are transient and do not severely limit the drug translocation. However, translocation of polar or charged drugs can be impacted by surface residues lining the interior of channel. We also noticed that the residues localized in the channel do not encompass a restrictive medium for the diffusion of water molecules inside the tunnel. We probed this, and Fig. 8C shows the calculated mean square displacement for water molecules inside the tunnel (red crosses) or in bulk water outside the pump (black circles). The total overlap shows that in either case the water encounters no restriction to its diffusion, suggesting that the tunnel does not affect water viscosity.

## Discussion and Conclusions

The interactions between MFPs and the outer membrane channels are essential for functionalities of efflux pumps and their versatility in complex assemblies. The spectrum of antibiotic resistance for non-cognate efflux systems was found to be extended by amino acid substitutions in the periplasmic component, particularly in either the α-hairpin^32,82^ that interacts with the outer membrane component, or the periplasmic β-barrel domain that interacts with the inner membrane component^82^. These results suggested that the proper alignment of MFPs with the two other components of the complex are critical for efflux function. Given the experimental demonstration that non-cognate assemblies can be obtained^32,35,82^, there might be a general mechanism triggering tripartite assembly but the assembly itself is not sufficient to enable effective efflux. This warrants a detailed study of the atomistic details of these interfaces, as well as their dynamics that can inform on potential activation mechanisms.

In the past, two models have been proposed to describe the mechanism of interaction between the periplasmic and outer membrane components in Gram-negative bacteria. The first model showed that the α-hairpins of the periplasmic component partially wrapping around and embedding the α-hairpins of the outer membrane protein^54^. The second model showed the inter-digitation of the α-hairpins from both components to give a cogwheel-like structure, and is commonly called the “tip-tip” model^53,56^. Cryo-EM studies of the intact tripartite pumps have been published supporting either of these models^53,54,56^. The most recent near-atomic resolution cryo-EM structures of AcrAB-TolC include apo (6.5-Å resolution), drug-bound (puromycin-bound at 5.9-Å resolution), and inhibitor-bound (MBX3132-bound at 3.6-Å resolution) structures that revealed the inactive, active, and inhibited states of this pump, respectively, and provide support for the “tip-tip” model^56^. Although “tip-tip” interactions between AcrA and TolC are seen in these recent cryo-EM structures, one noticeable difference is that TolC is closed at its periplasmic aperture in the apo structure while both ligand-bound structures showed opening of the TolC periplasmic aperture accompanied by more extensive inter-digitation between the AcrA and TolC α-hairpins. The structural and functional studies converged on the model in which binding of a drug molecule to one of the AcrB protomers in the apo state induces quaternary structural changes in AcrB that are transmitted to the AcrA hexamer to seal any gaps between its protomers, which in turn induces allosteric changes in TolC to open its periplasmic side and thereby create a continuous open channel involving AcrA and TolC^56,83,84^.

The experimental constraints (e.g., chemical crosslinks and/or fusions between monomers, chimeric constructs) used for obtaining the various cryo-EM structures for either of the two interaction models, however, raises issues of how functional these constructs are compared to the native complexes. As a first attempt to address this concern, Daury *et al*.^35^ provided the first reconstitution of native self-assembled Gram-negative RND pumps (*E*. *coli* AcrAB-TolC and *P*. *aeruginosa* MexAB-OprM) using synthetic nano-lipid platforms, and suggested that the assembly of tripartite RND pumps most likely follows a common mechanism. In addition, this study also reinforced the observed relative arrangement of pump components, showing that the periplasmic region bridges the inner and outer membrane components. This role of MFPs in the assembly process has also previously been investigated via SPR experiments^9^, showing that interactions between outer membrane and periplasmic components have higher affinity and are very critical for pump activation and drug translocation. Moreover, diverse levels of molecular complementarity between these two components are necessary for a fully functional pump. For instance, it has been observed that while different chimeric constructs can be self-assembled *in vitro* ^35,84^ with a median life time comparable to native pumps, their translocation activity is either weaker than that of native pumps or such assemblies are non-functional.

Our simulations provide relevant data for understanding the stability and complementarity process of the MexAB-OprM pump of *P*. *aeruginosa*. To our knowledge, our simulations are the first attempt to atomistically describe the dynamics of a fully assembled MDR pump in a Gram-negative cell envelope model. The initial atomistic structure (pre-MD simulations) obtained from our data-driven approach^70^ followed by an extensive molecular fitting^71^ to the available cryo-EM density map proved to be an excellent pipeline for building the atomic level structure of this tripartite pump. We note that the low-resolution cryo-EM map of AcrAB-TolC that we used for fitting shows a density for the TolC periplasmic aperture that is consistent with an open state (Figure S9), which would account for the OprM periplasmic aperture already being open in our fitted MexAB-OprM model. The subsequent MD simulation of intact MexAB-OprM pump in explicit membrane/water system shows that the interactions between the different components of the pump are stable within the timescale (1 μs) of molecular dynamics. The dynamics clearly show rearrangement of secondary structural elements of the complex, particularly those involved in the interaction between the periplasmic MexA and the outer membrane OprM. Although we are not directly accounting for the drug-dependent activation mechanism (LTO transitioning) of MexB in this study, our MD simulations reveal that the complementarity between the α-hairpins of the periplasmic hexamer (in a drug-bound-like conformation) and the outer membrane protein is sufficient for the engagement and opening of the latter.

Although we used the adaptor-wrapping cryo-EM structure of *E*. *coli* AcrAB-TolC pump^54^ for initially setting up our *P*. *aeruginosa* model, both the MDFF approach and unbiased MD simulations converged to a structure resembling the “tip-tip” model. The fact that the recent high-resolution cryo-EM structures of inactive and active AcrAB-TolC^56^ also show a “tip-tip” model with cogwheel-like interactions between the α-hairpins of AcrA and TolC provides strong support for our observations here regarding the MexA-OprM interface. Moreover, our sequence covariation analysis also provides support for the cogwheel configuration. The top ten predicted covarying and interacting pairs have residues that are near each other at the MexA-OprM interface using the “tip-tip” model (Figure S10A; see also plots in Fig. 3B and Figure S5), while they are farther from each other using the first model (Figure S10B,C). We note that the predicted interacting residue pairs from sequence covariation analysis are also consistent with those pairs identified from our MD simulations of the MDFF-fitted MexAB-OprM efflux pump. To our knowledge, this is the first application of sequence covariation analysis for cross-validating the interface contacts observed in simulations of a cryo-EM-fitted protein complex.

The sequence covariation analysis was also used to predict interacting residues in the *P*. *aeruginosa* TriABC-OpmH efflux complex^85^. In this efflux pump, two different MFPs (TriA and TriB) are required for the efflux of biocides such as triclosan and sodium dodecyl sulfate. These two MFPs are not interchangeable in their functions during drug efflux, with TriB alone being responsible for the opening of the outer membrane channel OpmH. Cysteine mutations at TriB residues E122, R126, or S127 were found to form disulfide bonds with cysteine mutations at OpmH residues E173 or I392^85^, and interestingly the homologous residues in MexA (L122, D126, Q127) and OprM (R211, K419) were all identified to be part of the MexA-OprM binding interface in our structural model (see Fig. 3). In addition, the sequence covariation analysis was able to identify two of the homologous MexA-OprM pairs, D126-K419 and D126-R211 (rank 1 and 4; see Table 1), as covarying and interacting pairs. We note that the disulfide interactions between the OpmH I392C mutant and any of the three TriB cysteine mutants were found to stabilize the open state of the channel as demonstrated by the leakage of azithromycin into cells^85^. Residue I392 is found on helix H8 of OpmH, and our simulations here similarly showed that the opening and closing of OprM is defined by the swinging of its helix H8 as it interacts with MexA.

It is also striking that the engagement mechanism for OprM observed in our simulations are fully supported by recent structural data^56^. Starting with a set of well packed periplasmic protomers resulting from LTO transitioning of MexB^68^, the direct interaction between MexA (hexamer) in a drug-bound-like conformation and OprM (trimer) triggers the distention of the periplasmic α-hairpins of OprM. The OprM aperture through our rotational enforced simulation is able to mimic the iris-like model proposed elsewhere^49,56^, suggesting that the activation is energetically favorable at reasonable timescales. This rotational opening of the outer membrane component in the counterclockwise direction (viewed from the periplasmic to extracellular) is consistent with the proposed conformational cycle of drug-proton antiport by the inner membrane component in the counterclockwise direction (also viewed from the intracellular to periplasmic)^68^. We should highlight, however, that further atomistic details on the allosteric transduction of the internal MexB transition to MexA are hard to extrapolate. Additional studies will be needed to address this part of the activation process to fully understand drug translocation by RND transporters.

The internal channel of the assembled pump (from docking domain to extracellular) proved to behave more like a diffusive tunnel for the translocation of small molecules. As demonstrated by our energetic calculations, the free energy for translocation falls within the expected thermal fluctuations of the system even for larger-size drugs like rifampicin. Thus, it is expected that the building-up of a chemical gradient (drug concentration) in the vestibule region should suffice to provide direction for drug extrusion. For instance, our calculations suggest that the central cavity may be able to host 40 rifampicin molecules, which translates to a 0.5 M concentration. Even with only a single drug molecule localized in this region, the concentration is still high enough compared to the extracellular space to drive efflux. A similar mechanism has been proposed for the MacAB-TolC ABC transporter^61^, in which a chemical gradient may be able to preferentially shuttle the drug from the inside to the outside of the pump. However, there are two major differences between the recent cryo-EM structures for AcrAB-TolC and MacAB-TolC: first, the apo cryo-EM structure of the latter has an open periplasmic aperture interacting with α-hairpins of the MacA hexamer (while this aperture is closed in the apo AcrAB-TolC cryo-EM structure); and second, there is a cluster of glutamine residues from the lipoyl domains of the MacA hexamer that act as a gating ring to control the movement of substrates within the channel (in contrast to opening/closing of the outer membrane protein periplasmic aperture acting as the gate).

In summary, we were able to build an intact model of MexAB-OprM pump using a combination of data-driven modeling and molecular fitting into cryo-EM data. Next, long timescale all-atom MD simulations of this structural model embedded in a Gram-negative membrane model were carried out. This fully assembled tripartite pump was stable within the boundaries of two-membrane and periplasmic space. We note that while our simulations here showed the efflux pump to be stable in POPC model lipid bilayers, future simulations should model the Gram-negative bacterial membrane in more detail. In particular, recent advancements in force field parametrizations of lipopolysaccharides (LPS)^86^ and peptidoglycan^87^ should allow for these components to be included in computational models and simulations. Using coarse-grained MD simulations, we then profiled the drug translocation from the docking domain of the MexB transporter, via the MexA hexameric and OprM α-domain and β-barrel conduit, towards the external medium. Importantly, we were able to characterize and refine the interface contacts, especially between MexA and OprM. An independent sequence covariation analysis confirmed these interfacial contacts based on evolutionary context. More detailed mechanistic studies of the MexA-OprM interface indicated that OprM is more than just a conduit, as its assembly with MexA in a drug-bound-like conformation is required to open the OprM periplasmic aperture and make the entire pump functional. Although past studies have accounted for the drug-dependent activation mechanism (LTO transitioning) of MexB leading to engagement of OprM, our studies reveal that it needs to be mediated by the interaction between the periplasmic and outer domains towards the opening of the latter. Key residues that take part in this mechanistic process, identified from the sequence and structural analyses, provide new strategies to interfere with the efflux pump assembly at the MexA-OprM interface.

## Methods

### Setup and MD simulations of all-atom systems

We first built an initial all-atom model of the fully assembled MexAB-OprM efflux pump via geometric simulations with FRODAN^88^ using the crystal structures of MexB (PDB 2V50)^44^, MexA (PDB 1VF7)^47^, and OprM (PDB 1WP1)^49^, as described in Phillips *et al*.^70^. This initial model was then fit into the 16-Å cryo-EM density map of apo AcrAB-TolC^54^ using the MD flexible fitting (MDFF) approach^71^, where the interactions between particles are described using the CHARMM force field. This fitted model of the MexAB-OprM pump was then embedded in a pre-equilibrated double bilayer system composed of 1-palmitoyl-2-oleoyl-sn-glycero-3-phosphocholine (POPC) lipids, with the transmembrane regions of OprM and MexB inserted into their respective bilayers using the inflateGRO algorithm^89^. This system comprised a total of 1.261 million particles, containing 1642 POPC lipids, 45 counter ions, and 315814 water molecules. Protein atoms were represented using the Amber ff99SB-ILDN force field^90^, POPC lipids were represented using the Amber Lipid14 force field^91^, and solvent was represented using the TIP3P model^92^.

To study the dynamics of OprM, the crystal structure of OprM that has a closed periplasmic aperture (PDB 1WP1)^49^ was inserted into a POPC bilayer using the inflateGRO algorithm^89^. This system comprised a total of 416000 particles, containing 694 POPC lipids, 25 counter ions, and 122325 water molecules. To study the MexA-dependent mechanism of OprM periplasmic aperture opening, we built two docked structures using the interactive protein docking software Hex^93^. The first structure involved docking of closed state OprM (PDB 1WP1)^49^ onto the MexA hexamer that was obtained earlier from MDFF fitting, and the second structure involved docking of closed state OprM (PDB 1WP1)^49^ onto the asymmetric MexB trimer (PDB 2V50)^44^. Both docked structures were placed in triclinic simulation boxes containing 49882 water molecules and ions to neutralize the net charge of each system. To study more closely this opening mechanism, we applied an enforced rotation procedure^94^ on the docked structure of closed OprM and MDFF-fitted MexA. Backbone atoms of residues in the OprM β-barrel region (R87-L99, A110-E125, I300-T308, and G322-F335) were positionally restrained during production runs to prevent rotation of this transmembrane region. The rotation of the MexA hexamer relative to OprM was enforced in either the clockwise or counter-clockwise direction, using a force constant of 400 kJ mol^−2^ and a rotation rate of 0.0001 degrees ps^−1^.

All MD simulations (except for the MDFF fitting) were performed using GROMACS v4.6.5^95^. Non-bonded interactions were handled using a twin-range cutoff scheme, with short-range and long-range cutoffs of 0.9 nm and 1.4 nm, respectively. All bond lengths were constrained with the LINCS algorithm^96^, allowing for a simulation time step of 2 fs. Long-range electrostatic contributions were computed using the PME approach^97^. System temperatures were maintained at 303 K by coupling to a velocity-rescaling thermostat^98^. System pressures were maintained at 1.0 bar using the Berendsen^99^ and Parrinello-Rahman^100^ barostats for equilibration and production runs, respectively. Membrane-embedded systems used semi-isotropic pressure coupling along the XY plane (i.e., the membrane plane), while non-membrane-embedded systems used isotropic pressure coupling. Prior to each production MD simulation, the system was energy minimized and equilibrated for 200 ns with position restraints on the protein backbone atoms. These restraints were then released for the production runs, with each trajectory covering 1 µs of simulation time. More details on the all-atom system setups and MD simulations can be found in the Supporting Information.

### Setup and MD simulations of coarse-grained systems

To compute the potential of mean force (PMF) of translocating antibiotic molecules through the MexAB-OprM tunnel, the MDFF-fitted model of this tripartite pump from earlier that had undergone equilibration was converted from an atomistic to a coarse-grained (CG) representation. The proteins, lipids and rifampicin drug were represented using the MARTINI model^101^, while the solvent was represented using a polarizable MARTINI water model^102^. CG parameters for rifampicin were calibrated against all-atom MD simulations and experimental octanol-water partition coefficients (logP_ow_). For this CG setup, the PMF of rifampicin translocation through the pump channel was computed using umbrella sampling^103^. A total of 220 windows were spaced 1 Å apart along the channel axis, with a restraining potential of 1000 kJ mol^−1^ nm^−2^ applied to the rifampicin center of mass. Each window was simulated for 1 µs, which were used for reconstructing the PMF via the weighted histogram approach^104^. Convergence was assessed by averaging the trajectories from 10 independent blocks of 0.1 µs each. More details on the CG system setups and MD simulations, particularly the calibration of CG parameters for rifampicin, can be found in the Supporting Information.

### Sequence covariation analysis between MexA and OprM

Two online tools were used for predicting residue pair interactions between MexA and OprM: GREMLIN (http://gremlin.bakerlab.org)^76^ and EVComplex (http://evcomplex.hms.harvard.edu)^77^. Both tools make predictions based on the principle that co-evolving protein partners generally show covariation between residues that are directly interacting. Full sequences of MexA and OprM were taken from UniProt^105^ entries P52477 and Q51487, respectively, and used as input into both online tools. For GREMLIN, the default E-value of 1e-20 was used for filtering BLAST search matches to each input sequence, and the default HHblits^106^ method was used for generating multiple sequence alignments (MSA). For EVComplex, the default E-value of 1e-5 was used for filtering BLAST search matches as well as the default jackhammer^107^ method for MSA generation. Prior to identifying covarying residue pairs between the two MSAs, both tools first use an ‘operon-based’ model to form concatenated sequence pairs that effectively combine the two MSAs. This model is particularly suited for analysis of prokaryotic proteins, as it assumes that co-regulated genes, and thus covarying proteins, are most likely to be near each other in the genome (e.g., such as for bacterial operons). Both tools therefore concatenate sequences that are closest in terms of genomic location. The final number of concatenated sequences used for subsequent residue pair scoring were 6008 and 4894 for GREMLIN and EVComplex, respectively. The top ten predicted covarying residue pairs from both tools were then compared and used for further analysis of the MexA-OprM interface from the 1-μs all-atom MD simulation of the MexAB-OprM complex. In particular, the inter-Cα and SC-COM distances for these ten residue pairs were measured for the last 500 ns of the simulation. Since each residue pair occurs three times in the full complex structure (due to the trimeric nature of OprM), cumulative averages for these two distance measures were computed over the three instances of each residue pair. Separate SC-COM distance averages were also computed for each residue pair instance.

### Data availability

The datasets generated during and/or analyzed during the current study are available from the corresponding author on reasonable request.

## Electronic supplementary material

Movie S1

Supporting Information
