# Supplementary material for: Dynamics of Intact MexAB-OprM Efflux Pump: Focusing on the MexA-OprM Interface
Source: Sci Rep. 2017 Nov 28;7:16521. doi: 10.1038/s41598-017-16497-w (PMC5705723; doi:10.1038/s41598-017-16497-w)
Supplement: Supplementary file 2 — Supporting Information [file 41598_2017_16497_MOESM2_ESM.pdf]

Supporting information

Dynamics of Intact MexAB-OprM Efflux Pump: Focusing on the MexA-OprM Interface

Cesar A. López, Timothy Travers, Klaas M. Pos, Helen I. Zgurskaya, and S. Gnanakaran

Supporting Methods

System setup of an intact efflux pump in a Gram-negative membrane model. We built the all-atom model of fully assembled *P. aeruginosa* MexAB-OprM based on fitting to the cryo-electron microscopy (cryo-EM) data of *E. coli* AcrAB-TolC solved by Du *et al.* [1] Initial coordinates for the fully assembled pump were obtained using geometric simulations with FRODAN [2] and adapted from Phillips *et al.* [3] These geometric simulations were performed using the crystal structures of MexB (PDB 2V50) [4], MexA (PDB 1VF7) [5], and OprM (PDB 1WP1) [6]. The coordinates for the MexB component corresponded to an asymmetric homotrimer that, while not drug-bound, had a detergent molecule in the multidrug-binding cavity of one of the protomers [4]. This detergent molecule was removed for the rest of the modeling done here. We note that the MexAB-OprM structural model obtained from FRODAN showed unfolding within the α-hairpin domains of MexA, which is because the geometric simulations targeted the shorter α-hairpins of MexA [7] into the longer α-hairpins of AcrA. To fix this, we decided to perform a rigid structural superimposition of the MexA crystallographic coordinates onto the FRODAN output, thereby replacing the partially-unfolded MexA prior to flexible fitting. This pump model was then fit into the cryo-EM map using the MD flexible fitting (MDFF) approach [8], with the α-carbon atoms used as the targets for fitting. Harmonic restraints were applied on the φ and ψ dihedral angles for residues within α-helices and β-sheets in order to properly retain these secondary structure elements seen in crystal structures of the individual pump components.

The low resolution of the cryo-EM map (16 Å) used for fitting may lead to partial deformation of regions with high uncertainty. In addition, the presence of unwanted molecules like detergents (specially localized in AcrB and TolC transmembrane regions) can mislead the orientation and further refinement of our model. Therefore, the MDFF fitting was applied here only to the entire MexA hexamer and to the periplasmic α-helical domains of the OprM trimer (including the interface between MexA and OprM), while position restraints were applied to the entire MexB trimer and to the transmembrane β-barrel domains of the OprM trimer (see **Figure 1**). The latter restraints were added in order to avoid any deformation of these components due to lack of density and to preserve the conformations that are specific to the crystal structures of MexB and OprM. During MDFF fitting, the interactions between particles are described using the CHARMM force field. The cryo-EM map is converted into a potential U_{md}, in which prohibited high energy states are allowed. A second term U_{ss} maintains the integrity of the secondary structure elements. Thus, during the MDFF procedure, the structure is biased towards the atomistic distribution found according to the cryo-EM density. A scaling factor of 0.3 kcal mol^{-1} was used during the fitting, which helps to avoid unwanted overfitting to the density map that can lead to distorted structures and unstable simulations. A total of a million fitting steps were necessary to observe convergence of the fitting (**Figure S1A**). More details about the flexible fitting methodology can be found in the original work [8].

The above initial model of MexAB-OprM efflux pump was posteriorly embedded in a fully hydrated double 1-palmitoyl-2-oleoyl-sn-glycero-3-phosphocholine (POPC) bilayer system, as shown in **Figure 2**. This system contained 1642 lipids, 45 counter ions

and 315814 water molecules, generating a total of 1.261 million particles. The protein was represented using the Amber ff99SB-ILDN [9], the POPC lipid was represented using the updated version Lipid14 of Amber [10], and water molecules were represented with the TIP3P model [11]. We used the inflateGRO algorithm [12] for embedding proteins within the double bilayer. Initially, the hydrophobic regions were detected by the voxel-based method [12]. Transmembrane regions in the OMF and RND were independently detected and used as targets to be embedded in the bilayers, as described in the original work [12]. Afterwards, two pre-equilibrated lipid patches were aligned with the hydrophobic regions and the fully assembled pump was inserted. Water molecules were then added such that these did not overlap with lipids and proteins. Before the production runs, the system was energy minimized and pre-equilibrated for 200 ns, restraining the backbone atoms of the protein and accounting for the membrane and water relaxation. Afterwards, restraints were released and the system was allowed to evolve according to Newton's equations.

All-atom system setup of OprM in POPC lipids. In order to study the dynamical properties of OprM, we embedded the non-gated (closed state) outer domain protein alone in a pure POPC bilayer. The initial coordinates for OprM were downloaded from the protein data bank (PDB 1WP1) [6]. Transmembrane regions in OprM were scanned as described for the case of the fully assembled pump and posteriorly inserted in a pre-equilibrated POPC bilayer using the inflateGRO method [12]. After protein insertion, the bilayer consisted of 694 POPC lipids. The system was solvated with 122325 water molecules and 25 counter ions were added in order to neutralize the excess of charges in the system. The backbone of the protein was positionally restrained in order to avoid any undesirable distortion of the protein during the equilibration phase (200 ns). The final system contained 416 K particles.

All-atom system setup of OprM-MexA and OprM-MexB complexes. We also probed the MexA-dependent aperture mechanism of the periplasmic tip of OprM by performing MD simulations using two different docked structures. The first configuration made use of the closed state of the OprM (PDB 1WP1) [6] and the MexA component that was obtained from the MDFF fitting. For the second configuration, the closed OprM component was placed in direct contact with the RND inner membrane transporter MexB (PDB 2V50) [4]. Configurations were obtained using the interactive protein docking and molecular superposition software Hex [13]. Briefly, Hex is an interactive molecular graphics program for the calculation of feasible docking modes of pairs of protein molecules. Each docked molecule is modeled using 3D expansions of real orthogonal spherical polar basis functions to encode both surface shape and electrostatic charge and potential distributions. The surface of proteins is represented using a two-term surface skin plus van der Waals steric density model, whereas the electrostatic model is derived from classical electrostatic theory. The different configurations obtained after the rigid docking approach are provided in **Figure S1A**. These configurations were placed in a triclinic box of 8.5x8.5x21 nm dimensions and fully solvated with 49882 water molecules. Ions were inserted to fully neutralize the excess charge of the system (15 Na^+ ions). Before the production runs, the atoms from the backbone were positionally restrained and equilibrated for 20 ns, allowing re-ordering of the surrounding water molecules. After relaxing the system, the constraints were released.

All-atom system setup of enforced OprM-MexA rotation. For the enforced rotational MD simulation, we used the docked MexA-OprM configuration (**Figure S1A**); thus, the structure containing the closed OprM domain and the MDFF-derived MexA domain was inserted in a box of 8.5x8.5x21 nm and fully solvated with 50000 water molecules. To avoid unwanted rotation of the transmembrane region of OprM, position restraints were applied to the backbone atoms corresponding to the beta-barrel region (R87-L99, A110-E125, I300-T308, G322-F335) embedded in the membrane. Before the production runs, the backbone atoms of the protein were kept in these positional restraints to allow the water molecules to fully relax. Later, only the membrane-embedded regions of OprM were restrained during the production run.

The enforced rotation procedure [14] applies a torque to a concerted set of atoms and has been successfully applied to several macromolecular systems [15]. The atoms of interest (rotation group) are subject to a rotational potential *V*. Each atom with position $\boldsymbol{x}_i$ gets assigned an equilibrium position $\boldsymbol{y}_i(t)$, rotating at a constant angular rate ω about an axis $\boldsymbol{v}$. The given initial positions of the selected atoms (rotation group) provide the starting configuration (*t=0*), which sets the reference positions $\boldsymbol{y}_i^0$. The potential assuring the rotation can be expressed as:

$$V^{iso} = \frac{k}{2}\sum_{i=1}^{N} W_i \left[\boldsymbol{\Omega}(t)(\mathbf{y}_i^0 - \mathbf{u}) - (\mathbf{x}_i - \mathbf{u})\right]^2 \quad (1)$$

The rotation matrix Ω and the pivot $\mathbf{u}$ of the axis yield forces towards the reference positions $\boldsymbol{y}_i(t)$, and effectively rotating $\boldsymbol{x}_i$. In our set up, we enforced the rotation of the MexA domain with respect of the OprM structure using a force constant (rot_k0) of 400 kJ mol^{-2}, and a rotation rate of 0.0001 degrees ps^{-1} either counter-clockwise or clockwise (see the Results section in the main text).

All-atom MD simulations. Molecular simulations (except the MDFF fitting) were carried out using the GROMACS molecular simulation package version 4.6.5 [16]. Simulations were performed using a 2 fs time step. The LINCS algorithm was applied to constrain all bond lengths with a relative geometric tolerance of 10^{-4} [17]. Non-bonded interactions were handled using a twin-range cutoff scheme. Within a short-range cutoff of 0.9 nm, the interactions were evaluated every time step based on a pair list updated every five-time steps. The intermediate-range interactions (up to a long-range cutoff radius of 1.4 nm) were evaluated simultaneously with each pair list update and were assumed constant in between. A PME approach [18] was used to account for electrostatic interactions with a grid spacing set to 0.15 nm. Constant temperature (303 K) was maintained by weak coupling of the solvent and solute separately to a velocity-rescaling scheme [19] with a relaxation time of 1.0 ps. During equilibration, the Berendsen algorithm [20] was used to couple the system pressure at 1.0 bar through an isotropic approach with relaxation time of 1.0 ps. After this steep, the pressure of the system was scaled using a Parrinello-Rahman barostat [21,22]. Membrane systems were quenched to 303 K and coupled using a semi-isotropic pressure approach within the X and Y plane. All systems were simulated as duplicates, except the larger fully assembled pump, which was run only once. Trajectories were run for 1 us and stored every 0.02 us for posterior analysis.

Coarse-grained system setup. In order to compute the Potential of Mean Force (PMF) of translocating antibiotic molecules through the assembled MDR tunnel, the equilibrated system from atomistic simulations was converted into a coarse-grained (CG) representation. The MARTINI model [23] was used to represent the protein, lipids, and the drug. The solvent was represented using the polarizable water model [24]. The drug was placed within the vestibule region of the assembled pump, and overlapping water molecules were removed before simulation.

The MARTINI force field contains a vast number of parameters for the simulation of proteins [25], lipids [23], carbohydrates [26], etc. However, the initial set of CG parameters for Rifampicin needs to be calibrated from all-atom MD simulations. In general, for a correct parameterization the MARTINI force field relies on a 4-to-1 mapping scheme and on the reproduction of partition coefficient between different solvents [23]. To obtain the CG coordinates of Rifampicin, an all-atom set-up of the molecule was obtained using the GAFF force field [27]. The molecule was equilibrated in a water box for 0.5 us and converted to a pseudo-CG trajectory using the center of mass of the most appropriate fine-grained particles [28]:

$$\mathbf{r}_i^{CG} = \frac{\sum_{j=1}^{p} \mathbf{r}_j . m_j}{\sum_{j=1}^{p} m_j} \qquad (2)$$

Where the vector $\mathbf{r}_i^{CG}$ describes the position of the pseudo-CG bead, p is the number of atoms mapped to a given coarse bead, m_j is the mass of the atom j, and $\mathbf{r}_j$ is its coordinates. From the atomistic simulation the target bonded distribution functions (bonds, angles, dihedrals) were obtained and iteratively introduced into the set of CG beads.

The coarse-grained models of antibiotic molecules were further improved by calibrating to experimental octanol-water ($\log P_{ow}$) partition coefficients. A closer inspection of the MARTINI bead types revealed that many chemical groups are already carefully parameterized matching experimental and calculated partitioning coefficients. Thus, bead selection was led by the most appropriate mapping scheme. The $\log P_{ow}$ was calculated as a difference between the solvation free energy in aqueous (ΔG_W) and octanol (ΔG_O) phase is the partitioning free energy ($\Delta\Delta G_{OW}$) [29]:

$$\Delta\Delta G_{OW} = -2.3RT\log P_{OW} \qquad (3)$$

where R and T correspond to the universal gas constant and the temperature of the system, respectively. ΔG_W and ΔG_O were calculated directly by uncoupling the non-bonded interactions of the solute with the respective solvent using the thermodynamic integration approach [30]:

$$\Delta F_{BA} = F_B - F_A = \int_{\lambda A}^{\lambda B} d\lambda \left\langle \frac{\partial U_{uv}(\lambda)}{\partial \lambda} \right\rangle \lambda \qquad (4)$$

Here λ is a coupling parameter that regulates the strength of the interaction of F_B (fully uncoupled) and F_A (fully coupled). $U_{uv}(\lambda)$ denotes the potential energy function describing the total solute-solvent interaction. The average $\langle \cdots \rangle$ is taken over the MD trajectory. Calculations were performed at 25 independent λ points. For each individual

λ, simulations were run for 50 ns (AA) or 100 ns (CG) respectively. After parameterization of the CG model, the obtained logP was 1.75, in good agreement with the experimental value (3.7) [31]. The final set of bonded and non-bonded terms for the coarse-grained representation are provided in the **Supporting Data** section.

Coarse-grained MD simulations. Briefly, the non-bonded interactions were cut off at a distance r_{cut} of 1.2 nm. To reduce the generation of unwanted noise, we used the standard shift function of GROMACS, in which both the energy and force smoothly vanish at the cutoff distance. The global dielectric constant was adjusted to ε=2.5. The LJ and Coulomb potentials were shifted from r = 0.0 and r = 0.9 nm to the cutoff distance (r = 1.2 nm), respectively. The time step used to integrate the equations of motion was 25 fs. Constant temperature at 303 K was maintained by weak coupling of the solvent and membranes separately to a Berendsen [20] heat bath with a relaxation time of 1.0 ps. Pressure was controlled using the Parrinello-Rahman barostat [21,22] set at 1.0 bar.

The drug translocation potential of mean force (PMF) by MDR was calculated using the umbrella sampling approach [32]. The simulation was composed of 220 independent points spaced 1 Å apart. A restraining potential of 1000 kJ mol^{-1} nm^{-2} was applied to the center of mass of the entire drug with respect of the center of mass of the inner membrane component (MexB domain). One micro-second long simulations per point was performed along the Z reaction coordinate. PMFs were reconstructed using the weighted histogram [33] approach, and convergence was assessed by averaging the trajectories from 10 independent blocks of 0.1 us each.

References

1 Du, D. *et al.* Structure of the AcrAB-TolC multidrug efflux pump. *Nature* **509**, 512-515, doi:10.1038/nature13205 (2014).

2 Farrell, D. W., Speranskiy, K. & Thorpe, M. F. Generating stereochemically acceptable protein pathways. *Proteins* **78**, 2908-2921, doi:10.1002/prot.22810 (2010).

3 Phillips, J. L. & Gnanakaran, S. A data-driven approach to modeling the tripartite structure of multidrug resistance efflux pumps. *Proteins* **83**, 46-65, doi:10.1002/prot.24632 (2015).

4 Sennhauser, G., Bukowska, M. A., Briand, C. & Grutter, M. G. Crystal structure of the multidrug exporter MexB from Pseudomonas aeruginosa. *J Mol Biol* **389**, 134-145, doi:10.1016/j.jmb.2009.04.001 (2009).

5 Akama, H. *et al.* Crystal structure of the membrane fusion protein, MexA, of the multidrug transporter in Pseudomonas aeruginosa. *J Biol Chem* **279**, 25939-25942, doi:10.1074/jbc.C400164200 (2004).

6 Akama, H. *et al.* Crystal structure of the drug discharge outer membrane protein, OprM, of Pseudomonas aeruginosa: dual modes of membrane anchoring and occluded cavity end. *J Biol Chem* **279**, 52816-52819, doi:10.1074/jbc.C400445200 (2004).

7 Xu, Y. *et al.* Funnel-like hexameric assembly of the periplasmic adapter protein in the tripartite multidrug efflux pump in gram-negative bacteria. *J Biol Chem* **286**, 17910-17920, doi:10.1074/jbc.M111.238535 (2011).

8 Trabuco, L. G., Villa, E., Schreiner, E., Harrison, C. B. & Schulten, K. Molecular dynamics flexible fitting: a practical guide to combine cryo-electron microscopy and X-ray crystallography. *Methods* **49**, 174-180, doi:10.1016/j.ymeth.2009.04.005 (2009).

9 Lindorff-Larsen, K. *et al.* Improved side-chain torsion potentials for the Amber ff99SB protein force field. *Proteins* **78**, 1950-1958, doi:10.1002/prot.22711 (2010).

10 Dickson, C. J. *et al.* Lipid14: The Amber Lipid Force Field. *J Chem Theory Comput* **10**, 865-879, doi:10.1021/ct4010307 (2014).

11 Jorgensen, W. L., Chandrasekhar, J., Madura, J. D., Impey, R. W. & Klein, M. L. Comparison of simple potential functions for simulating liquid water. *The Journal of Chemical Physics* **79**, 926-935, doi:doi:http://dx.doi.org/10.1063/1.445869 (1983).

12 Schmidt, T. H. & Kandt, C. LAMBADA and InflateGRO2: efficient membrane alignment and insertion of membrane proteins for molecular dynamics simulations. *J Chem Inf Model* **52**, 2657-2669, doi:10.1021/ci3000453 (2012).

13 Ritchie, D. W. & Venkatraman, V. Ultra-fast FFT protein docking on graphics processors. *Bioinformatics* **26**, 2398-2405, doi:10.1093/bioinformatics/btq444 (2010).

14 Kutzner, C., Czub, J. & Grubmuller, H. Keep It Flexible: Driving Macromolecular Rotary Motions in Atomistic Simulations with GROMACS. *J Chem Theory Comput* **7**, 1381-1393, doi:10.1021/ct100666v (2011).

15 Kumar, R. & Grubmuller, H. Elastic properties and heterogeneous stiffness of the phi29 motor connector channel. *Biophys J* **106**, 1338-1348, doi:10.1016/j.bpj.2014.01.028 (2014).

16 Pronk, S. *et al.* GROMACS 4.5: a high-throughput and highly parallel open source molecular simulation toolkit. *Bioinformatics* **29**, 845-854, doi:10.1093/bioinformatics/btt055 (2013).

17 Hess, B. P-LINCS: A Parallel Linear Constraint Solver for Molecular Simulation. *J Chem Theory Comput* **4**, 116-122, doi:10.1021/ct700200b (2008).

18 Darden, T., York, D. & Pedersen, L. Particle mesh Ewald: An N·log(N) method for Ewald sums in large systems. *The Journal of Chemical Physics* **98**, 10089-10092, doi:doi:http://dx.doi.org/10.1063/1.464397 (1993).

19 Bussi, G., Donadio, D. & Parrinello, M. Canonical sampling through velocity rescaling. *The Journal of Chemical Physics* **126**, 014101, doi:10.1063/1.2408420 (2007).

20 Berendsen, H. J. C., Postma, J. P. M., van Gunsteren, W. F., DiNola, A. & Haak, J. R. Molecular dynamics with coupling to an external bath. *The Journal of Chemical Physics* **81**, 3684-3690, doi:doi:http://dx.doi.org/10.1063/1.448118 (1984).

21 Parrinello, M. & Rahman, A. Crystal Structure and Pair Potentials: A Molecular-Dynamics Study. *Physical Review Letters* **45**, 1196-1199 (1980).

22 Nosé, S. & Klein, M. L. Constant pressure molecular dynamics for molecular systems. *Molecular Physics* **50**, 1055-1076, doi:10.1080/00268978300102851 (1983).
23 Marrink, S. J., Risselada, H. J., Yefimov, S., Tieleman, D. P. & de Vries, A. H. The MARTINI force field: coarse grained model for biomolecular simulations. *J Phys Chem B* **111**, 7812-7824, doi:10.1021/jp071097f (2007).
24 Yesylevskyy, S. O., Schafer, L. V., Sengupta, D. & Marrink, S. J. Polarizable water model for the coarse-grained MARTINI force field. *PLoS Comput Biol* **6**, e1000810, doi:10.1371/journal.pcbi.1000810 (2010).
25 Monticelli, L. *et al.* The MARTINI Coarse-Grained Force Field: Extension to Proteins. *J Chem Theory Comput* **4**, 819-834, doi:10.1021/ct700324x (2008).
26 Lopez, C. A. *et al.* Martini Coarse-Grained Force Field: Extension to Carbohydrates. *J Chem Theory Comput* **5**, 3195-3210, doi:10.1021/ct900313w (2009).
27 Wang, J., Wolf, R. M., Caldwell, J. W., Kollman, P. A. & Case, D. A. Development and testing of a general amber force field. *J Comput Chem* **25**, 1157-1174, doi:10.1002/jcc.20035 (2004).
28 Rzepiela, A. J. *et al.* Reconstruction of atomistic details from coarse-grained structures. *J Comput Chem* **31**, 1333-1343, doi:10.1002/jcc.21415 (2010).
29 Best, S. A., Merz, K. M. & Reynolds, C. H. Free Energy Perturbation Study of Octanol/Water Partition Coefficients: Comparison with Continuum GB/SA Calculations. *The Journal of Physical Chemistry B* **103**, 714-726, doi:10.1021/jp984215v (1999).
30 van Gunsteren, W. F. & Berendsen, H. J. Thermodynamic cycle integration by computer simulation as a tool for obtaining free energy differences in molecular chemistry. *J Comput Aided Mol Des* **1**, 171-176 (1987).
31 Rifampin. *Tuberculosis* **88**, 151-154, doi:http://dx.doi.org/10.1016/S1472-9792(08)70024-6 (2008).
32 Torrie, G. M. & Valleau, J. P. Nonphysical sampling distributions in Monte Carlo free-energy estimation: Umbrella sampling. *Journal of Computational Physics* **23**, 187-199, doi:http://dx.doi.org/10.1016/0021-9991(77)90121-8 (1977).
33 Hub, J. S., de Groot, B. L. & van der Spoel, D. g_wham—A Free Weighted Histogram Analysis Implementation Including Robust Error and Autocorrelation Estimates. *Journal of Chemical Theory and Computation* **6**, 3713-3720, doi:10.1021/ct100494z (2010).
34 Wang, Z. *et al.* An allosteric transport mechanism for the AcrAB-TolC Multidrug Efflux Pump. *Elife* **6**, doi:10.7554/eLife.24905 (2017).
35 Jeong, H. *et al.* Pseudoatomic Structure of the Tripartite Multidrug Efflux Pump AcrAB-TolC Reveals the Intermeshing Cogwheel-like Interaction between AcrA and TolC. *Structure* **24**, 272-276, doi:10.1016/j.str.2015.12.007 (2016).

Supporting Figures

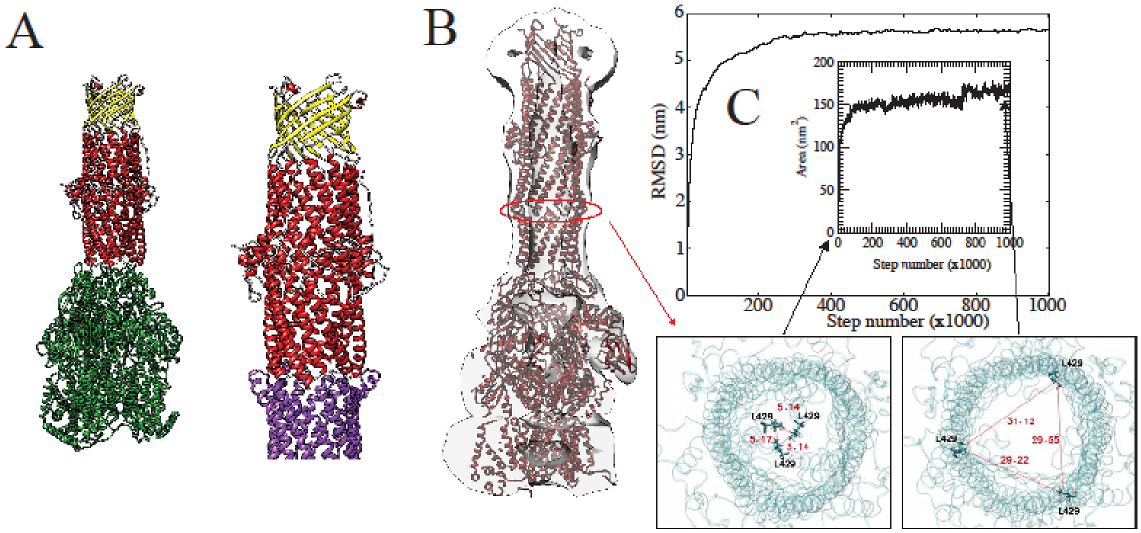

Figure S1. MDFF-based cryo-EM fitting of MexAB-OprM efflux pump. (A) Probed structures for the interaction between OprM-MexB (left) and OprM-MexA (right). Configurations were obtained by rigid docking using Hex (see **Supporting Methods**). (B) Overlap of the fully assembled pump using the 16-Å cryo-EM structure [1] as reference. (C) Plot showing the number of steps for convergence of the root mean square displacement (RMSD) of the backbone atoms and the density of the cryo-EM map. Small inset plot shows the variational aperture of the periplasmic tips of OprM as a function of the MDFF fitting steps. The area is defined as a triangular shape enclosed by L429. Structures are provided for the initial configuration (bottom left inset) and the final configuration (bottom right inset) with their respective distances.

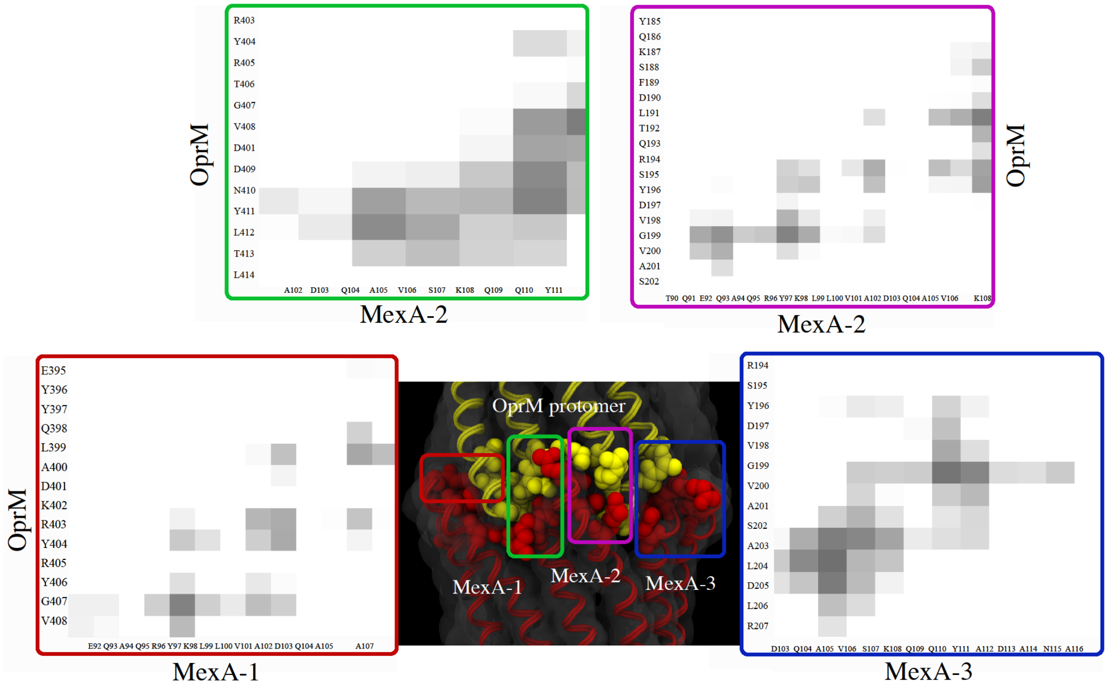

Figure S2. Contact maps for the different components of the MexA and OprM domains. Different colors refer to the different domains enclosed within squares.

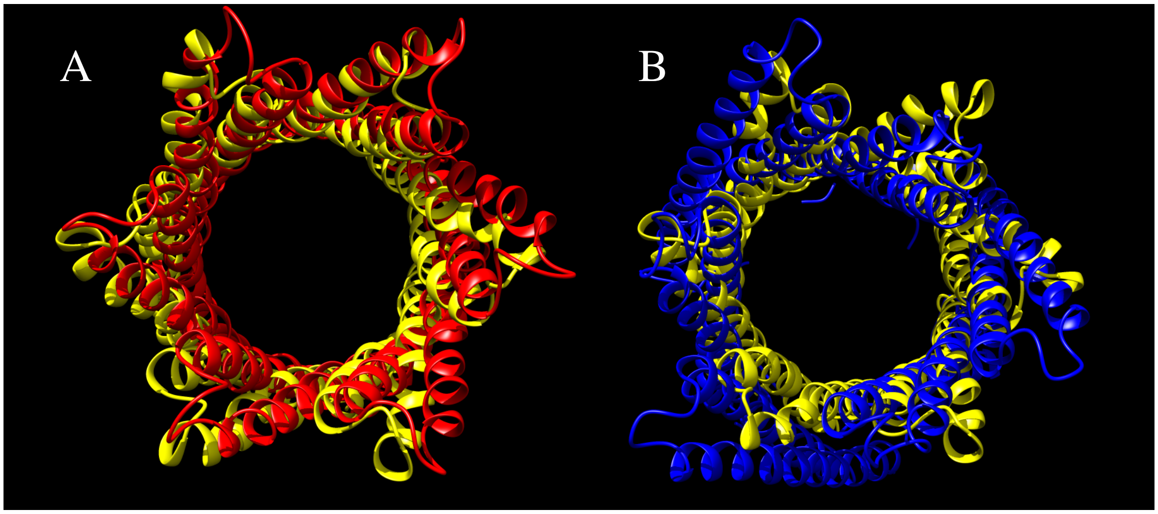

Figure S3. Structural overlaps of *P. aeruginosa* and *E. coli* periplasmic proteins. Our MDFF-fitted model of MexAB-OprM (yellow ribbons; fitted to the 16-Å cryo-EM map [1] of AcrAB-TolC) was compared with both the drug-bound (red ribbons in panel A; PDB 5NG5) and apo (blue ribbons in panel B; PDB 5V5S) models from the recent high-resolution cryo-EM maps [34] of AcrAB-TolC. This clearly shows that our MDFF fitting to the low-resolution cryo-EM map drove MexA closer to the configuration seen in the drug-bound state.

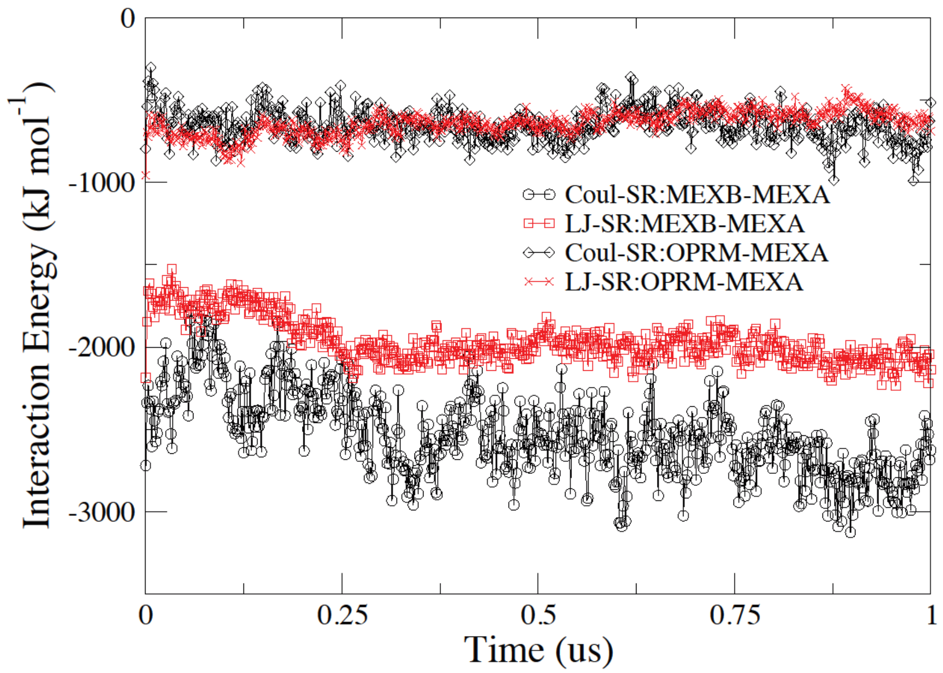

Figure S4. Interaction energy as a function of time for the MexAB-OprM pump MD simulation. Energies are decomposed as coulomb between MexB-MexA (black circles), Lennard-Jones between MexB-MexA (red squares), coulomb between OprM-MexA (black diamonds), and Lennard-Jones between OprM-MexA (red crosses).

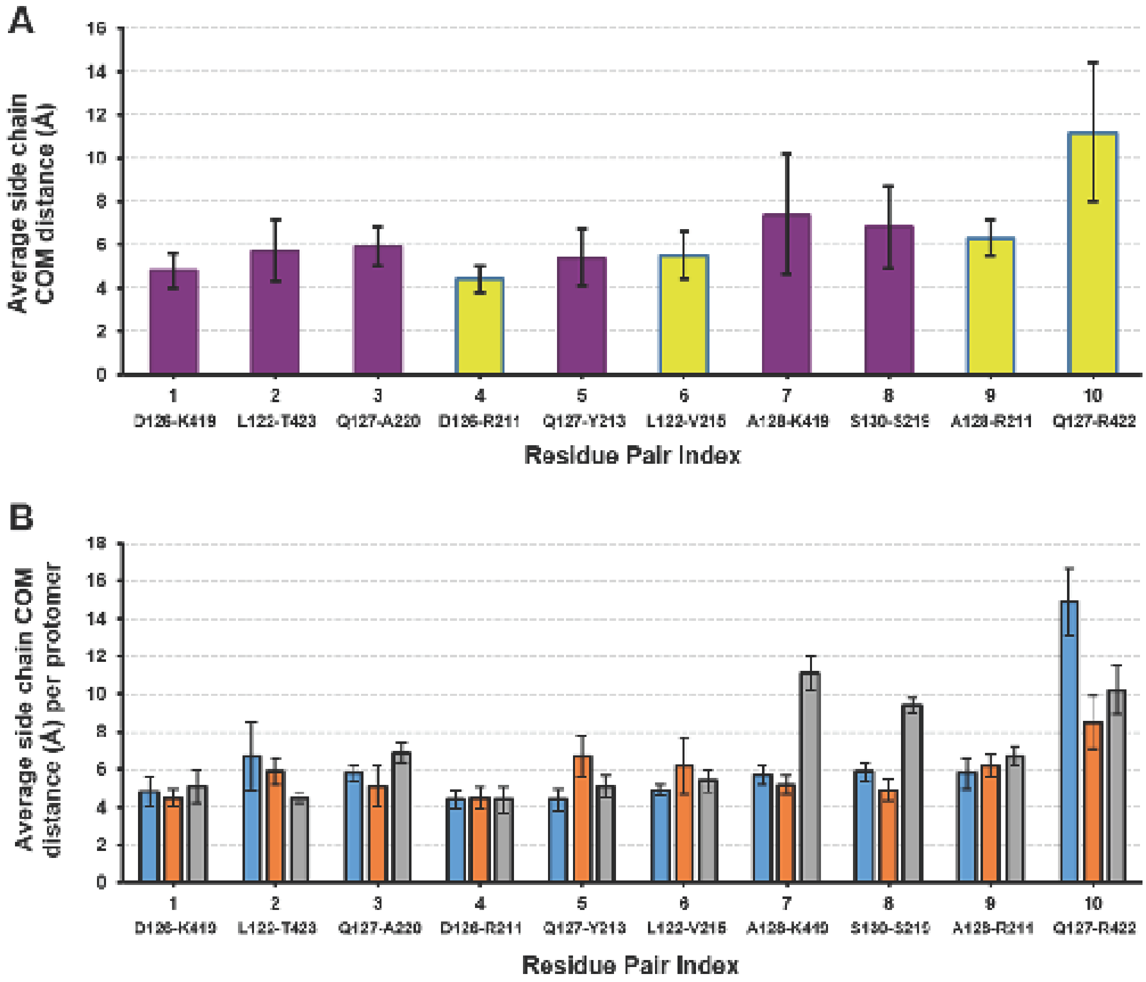

Figure S5. Measurement of simulation contacts for the ten predicted covarying residue pairs. (A) Average side chain center of mass (COM) distance for each of the ten predicted residue pairs over all three protomers in the last 500 ns of the 1-μs MD simulation of MexAB-OprM. Residue pairs are colored either violet or yellow depending to which of the two interfaces shown in **Figure 3A** these belong. (B) Breakdown by protomer of each overall average side chain COM distance in (A). All error bars give s.d.

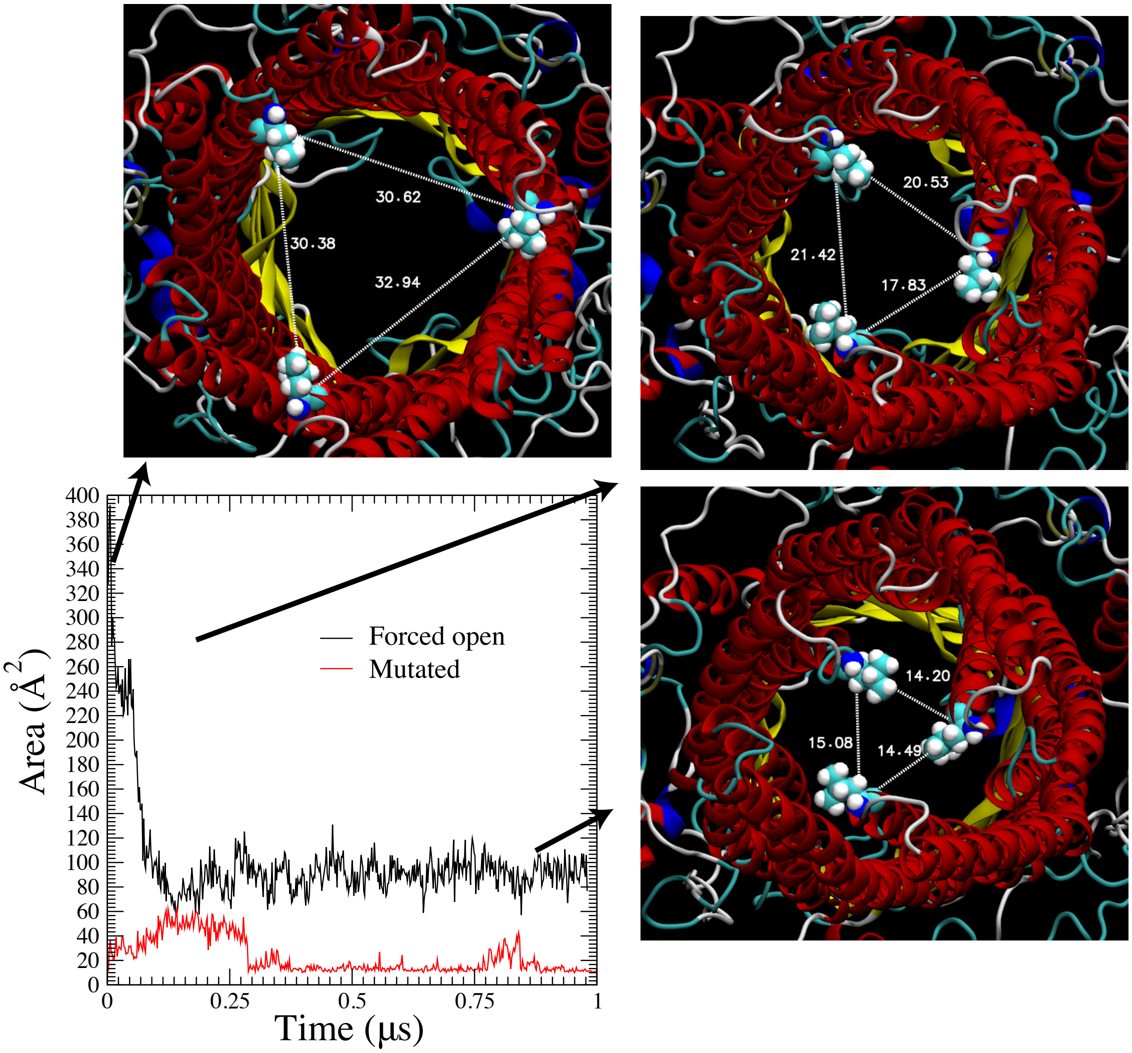

Figure S6. Probed aperture area of OprM for a forcefully opened OprM structure (black line) and a mutated OprM (red line). The insets highlight three states of the pre-relaxed OprM. The initial configuration of opened OprM is characterized by a maximum aperture (400 Å^2) area. However, as the simulation progressed, this area contracted to an equilibrated value of ~90 Å^2.

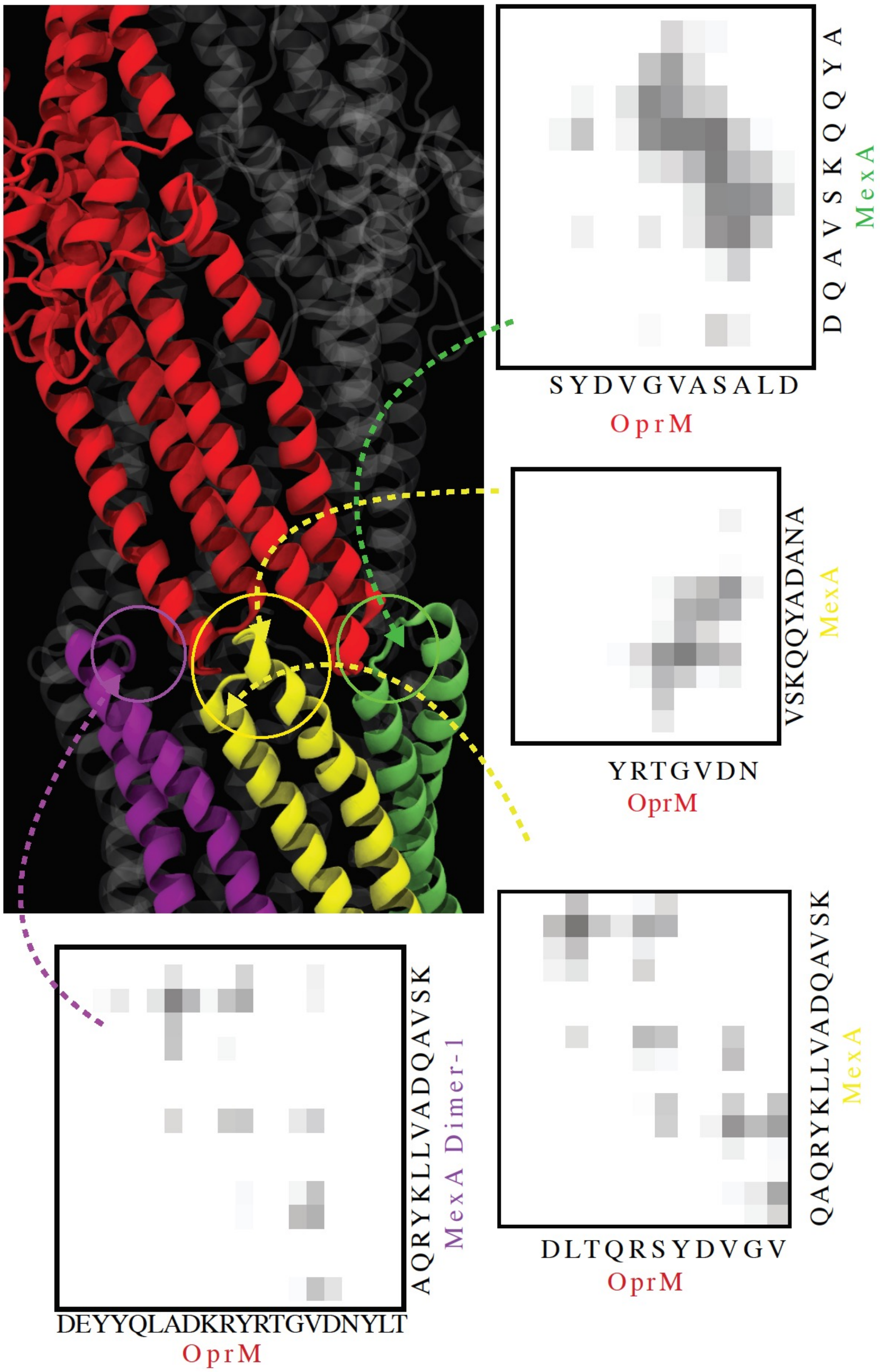

Figure S7. Residue contact maps between an OprM protomer and three adjacent periplasmic MexA proteins. The figure is an extension of **Figure 5** in the main text.

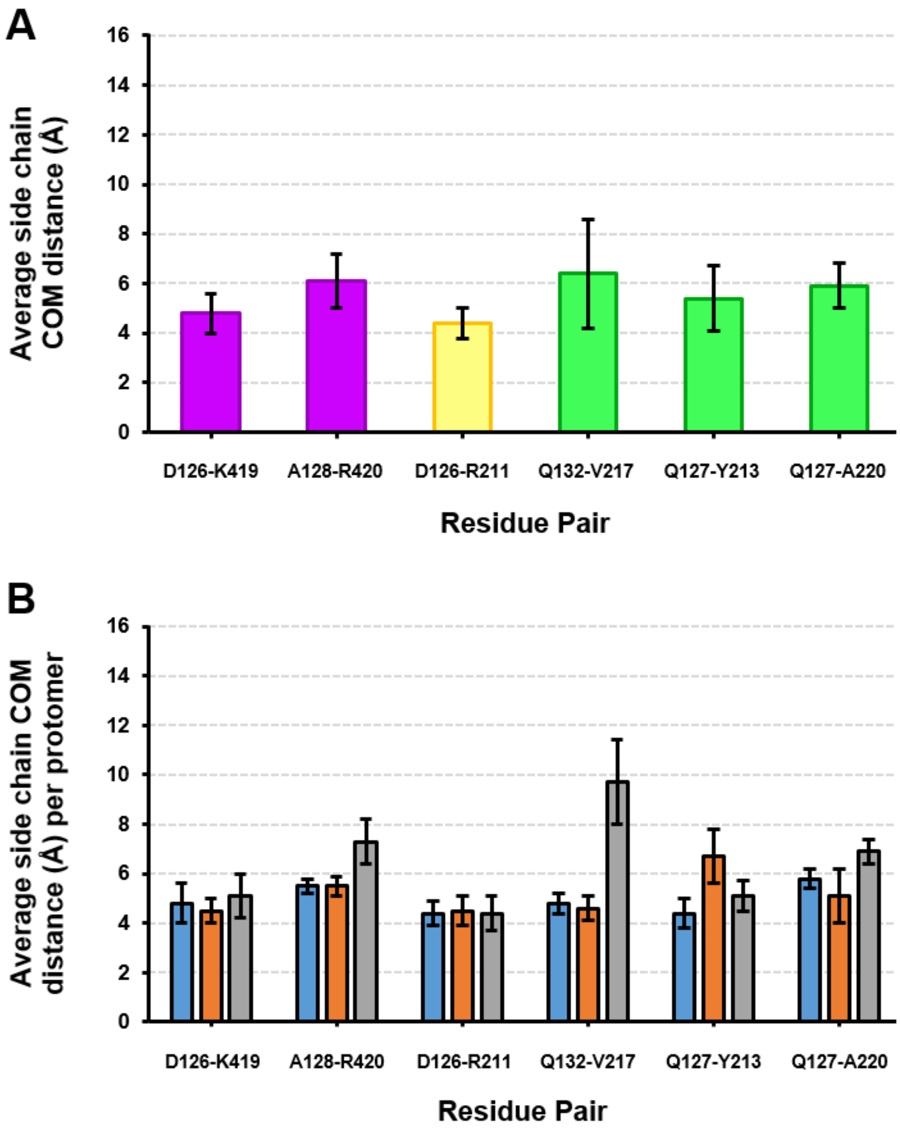

Figure S8. Measurement of simulation contacts for the interacting residue pairs observed during OprM activation (see Figure 5 of main text). (A) Average side chain center of mass (COM) distance for each of the six observed interacting residue pairs over all three protomers in the last 500 ns of the 1-μs MD simulation of MexAB-OprM. Residue pairs are colored either purple, yellow, or green depending to which of the three interfaces shown in **Figure 5** these belong. (B) Breakdown by protomer of each overall average side chain COM distance in (A). All error bars give s.d.

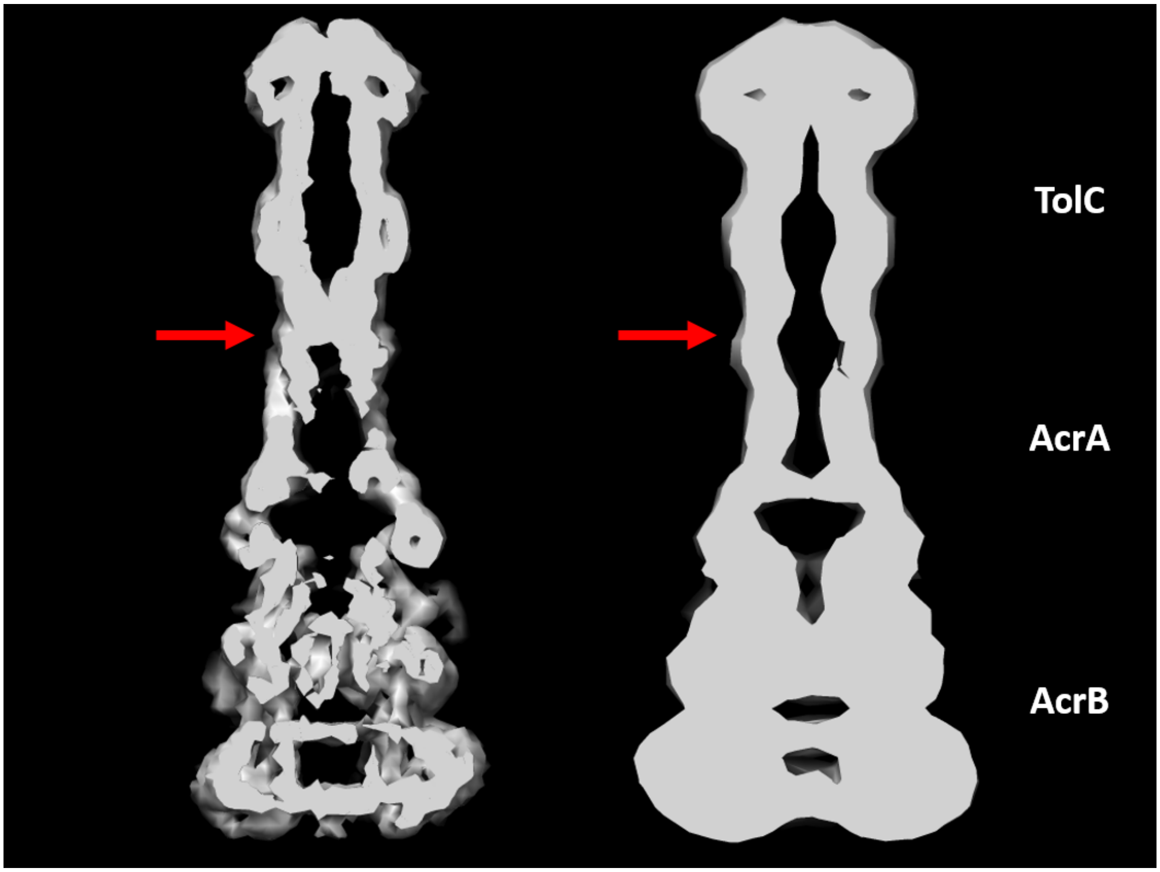

Figure S9. Comparison of cryo-EM density maps for apo AcrAB-TolC showing differences in closing/opening of the TolC periplasmic aperture (red arrows). Components of this tripartite efflux pump are labeled on the right. The density map on the left corresponds to the recent high-resolution (6.5 Å) cryo-EM structure [34], and shows the TolC α-hairpins adopting a closed periplasmic aperture. The density map on the right (which was used here for MDFF-based fitting of MexAB-OprM) corresponds to the earlier low-resolution (16 Å) cryo-EM structure [1], and shows the TolC α-hairpins adopting an open periplasmic aperture such that there is a continuous channel between the TolC trimer and AcrA hexamer.

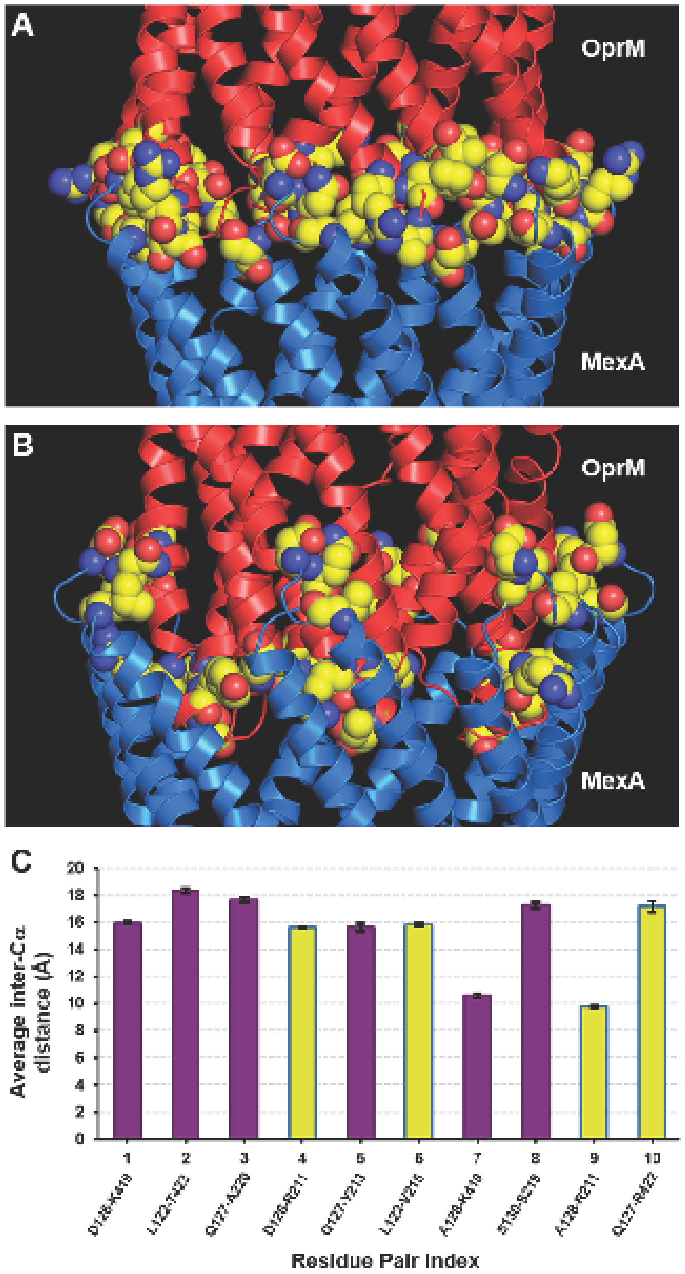

Figure S10. Mapping of the ten predicted residue pairs onto different atomistic models of the MexA-OprM interface. These models are based on the cryo-EM-fitted structures for AcrAB-TolC that describe either an (A) "adaptor-bridging" [34,35] or (B) "adaptor-wrapping" [1] interface. MexA and OprM monomers are shown as blue and red cartoons, respectively. The ten residue pairs are shown as atomic spheres, with carbon, oxygen, and nitrogen atoms colored yellow, red, and blue, respectively. (C) Average inter-Cα distances for each of the ten residue pairs over all three protomers in an "adaptor-wrapping" model of the MexA-OprM interface. Compare with the corresponding plot for the "adaptor-bridging" model shown in **Figure 3B**. Error bars give s.d.

Supporting Tables

MexB residue	OprM Residue	Score GREMLIN
V265	L39	0.0
V265	Y156	0.0
V61	G277	0.0
L251	T348	0.0
A12	A48	0.0

Table S1. Predictions of covarying residue pairs between MexB and OprM by GREMLIN. Gremlin scores are expressed as probabilities that predicted residue pairs are covarying and interacting, with values ≥ 0.70 giving high-confidence predictions. All the predicted residues pairs for MexB-OprM have scores of 0, and shown are five of these pairs.

Supporting Movies

Movie S1. MexA-dependent mechanism of iris-like opening for the OprM periplasmic aperture. Residues involved during the periplasmic aperture opening of OprM are labeled with different color codes (Orange – Leu429, Magenta – Tyr413, Lime – Asp433, Cyan – Arg436, and Pink – Ala392). OprM protomers are shown in cartoons with different colors (red, blue, and yellow). (Top-left) The counter-clockwise rotation features an increase in the area enclosed by the triplet of Leu429 residues (see **Results** in the main text). OprM is viewed from its periplasmic aperture. (Top-right) Changes in the potential of mean force (PMF) with increasing angle rotation are shown in order to visualize the force-dependent mechanism. (Bottom) The rearrangements leading to better cogwheel-like interactions between the OprM α-hairpins with those of MexA (depicted as translucent gray cartoons). OprM is viewed from its side.

Supporting Data

MARTINI CG parameters for the simulation of Rifampicin

```
[ moleculetype ]
; Name           nrexcl
RIFA           1

[ atoms ]
;   nr      type  resnr residue  atom   cgnr     charge       mass  typeB    chargeB      massB
    1        N0     1   RIFA     R1      1         0        72   ; qtot 0
    2       SC1     1   RIFA     R2      2         0        72   ; qtot 0
    3       SN0     1   RIFA     R3      3         0        72   ; qtot 0
    4        N0     1   RIFA     R4      4         0        72   ; N0 high pH, Qd at low pH and charge +1
    5        P2     1   RIFA     R5      5         0        72   ; qtot 0
    6        Na     1   RIFA     R6      6         0        72   ; qtot 0
    7        P2     1   RIFA     R7      7         0        72
    8        Na     1   RIFA     R8      8         0        72
    9       SC3     1   RIFA     R9      9         0        72
   10        N0     1   RIFA    R10     10         0        72
   11        N0     1   RIFA    R11     11         0        72
   12        N0     1   RIFA    R12     12         0        72
   13        C1     1   RIFA    R13     13         0        72
   14        Na     1   RIFA    R14     14         0        72
   15        P1     1   RIFA    R15     15         0        72
   16        P1     1   RIFA    R16     16         0        72
   17        C3     1   RIFA    R17     17         0        72
   18        C3     1   RIFA    R18     18         0        72
   19        P5     1   RIFA    R19     19         0        72

[ bonds ]
;  ai    aj funct           c0           c1           c2           c3
;#ifdef FLEXIBLE
   1     2        1 0.268 40000
   1     3        1 0.299 40000
   2     3        1 0.223 40000
   3     4        1 0.282 40000
   4     5        1 0.356 40000
   4     6        1 0.297 40000
   5     7        1 0.249 40000
   6     7        1 0.389 40000
   6     8        1 0.236 40000
   7     9        1 0.239 40000
   8     9        1 0.376 40000
   8    10        1 0.232 40000
   9    10        1 0.327 40000
  10    11        1 0.269 40000
  13    15        1 0.293 40000
  15    16        1 0.251 40000
  16    17        1 0.292 40000
  18    19        1 0.243 40000
;#endif
  11    12        1 0.333 40000
  12    13        1 0.280 40000
  13    14        1 0.362 40000
  17    18        1 0.394 40000
  19     5        1 0.230 40000

[ angles ]
1   3   4 2 156.00 500.00
2   3   4 2 101.00 280.00
4   5   7 2 125.00 800.00
4   6   7 2 102.00 500.00
5   7   9 2 150.00 900.00
4   6   8 2 168.00 300.00
7   6   8 2 88.00  700.00
```

```
7   9   8 2 91.00 600.00
7   9  10 2 128.00 900.00
6   8   9 2 92.00 600.00
6   8  10 2 150.00 150.00
9   8  10 2 60.00 400.00
9  10   8 2 82.00 500.00
9  10  11 2 91.00 200.00
8  10  11 2 74.00 250.00
10 11  12 2 145.00 300.00
11 12  13 2 80.00 400.00
12 13  14 2 72.00 130.00
12 13  15 2 129.00 300.00
13 15  16 2 144.00 270.00
14 13  15 2 59.00 220.00
15 16  17 2 117.00 150.00
16 17  18 2 140.00 150.00
17 18  19 2  95.00 150.00
18 19   5 2  145.00 250.00
19  5   4 2 68.00 500.00
19  5   7 2 160.00 20.00

[ exclusions ]
2 4
4 7
4 8
5 9
5 6
5 8
6 9
6 10
7 8
7 10
9 11
8 11
10 12
11 13
12 14
14 15
15 17
16 18
19 4
19 7

;#ifndef FLEXIBLE
[ constraints ]
;   1     2        1 0.268
;   1     3        1 0.299
;   2     3        1 0.223
;   3     4        1 0.282
;   4     5        1 0.356
;   4     6        1 0.297
;   5     7        1 0.249
;   6     7        1 0.389
;   6     8        1 0.236
;   7     9        1 0.239
;   8     9        1 0.376
;   8    10        1 0.232
;   9    10        1 0.327
;  13    15        1 0.293
;  15    16        1 0.251
;  16    17        1 0.292
;  18    19        1 0.243
;#endif
```
